# Drug-controlled CAR T cells through the regulation of cell–cell interactions

**DOI:** 10.1038/s41589-026-02152-x

**Published:** 2026-02-19

**Authors:** Leo Scheller, Greta Maria Paola Giordano Attianese, Rocío Castellanos-Rueda, Raphaël B. Di Roberto, Markus Barden, Melanie Triboulet, Morteza Hafezi, Sarah Ash, Sailan Shui, Elisabetta Cribioli, Patrick Reichenbach, Jimmy Maillard, Anthony Marchand, Sandrine Georgeon, Benita Wolf, Hinrich Abken, Sai Reddy, Bruno E. Correia, Melita Irving

**Affiliations:** 1 Laboratory of Protein Design and Immunoengineering, École Polytechnique Fédérale de Lausanne (EPFL), Lausanne, Switzerland; 2 Structural Biochemistry Group, Leibniz Institute for Immunotherapy, Regensburg, Germany; 3 Ludwig Institute for Cancer Research, Department of Fundamental Oncology, University of Lausanne, Lausanne, Switzerland; 4 Department of Biosystems Science and Engineering, Swiss Federal Institute of Technology in Zürich (ETH Zürich), Basel, Switzerland; 5 Life Science Zürich Graduate School, Swiss Federal Institute of Technology in Zürich (ETH Zürich), University of Zürich, Zurich, Switzerland; 6 Division of Genetic Immunotherapy, Leibniz Institute for Immunotherapy, Regensburg, Germany; 7 Lausanne University Hospital (CHUV), Lausanne, Switzerland

**Keywords:** Cancer therapy, Screening

## Abstract

Chimeric antigen receptor (CAR) T cell therapy is constrained by on-target, off-tumor toxicities and cellular exhaustion because of chronic antigen exposure. CARs incorporating small-molecule controlled on- and off-switches can enhance both safety and therapeutic efficacy but their design is limited by the scarcity of nonimmunogenic protein elements responsive to nonimmunosuppressive, clinically approved drugs with favorable pharmacodynamics. Here we combine rational design and library-based optimization of a protein–protein interaction (PPI) of human origin to develop venetoclax-controlled drug-regulated off-switch PPI (DROP)-CARs. DROP-CARs enable dose-dependent release of the tumor-targeting scFv and consequent reduction in T cell binding to the tumor cell. Additionally, we present proof of concept for a dual DROP-CAR controlled by different small molecules, as well as for logic-gated synthetic receptors enabling STAT3 signaling. We demonstrate in vitro and in vivo function of DROP-CAR T cells and conclude that the approach holds promise for clinical application.

**Figure:**
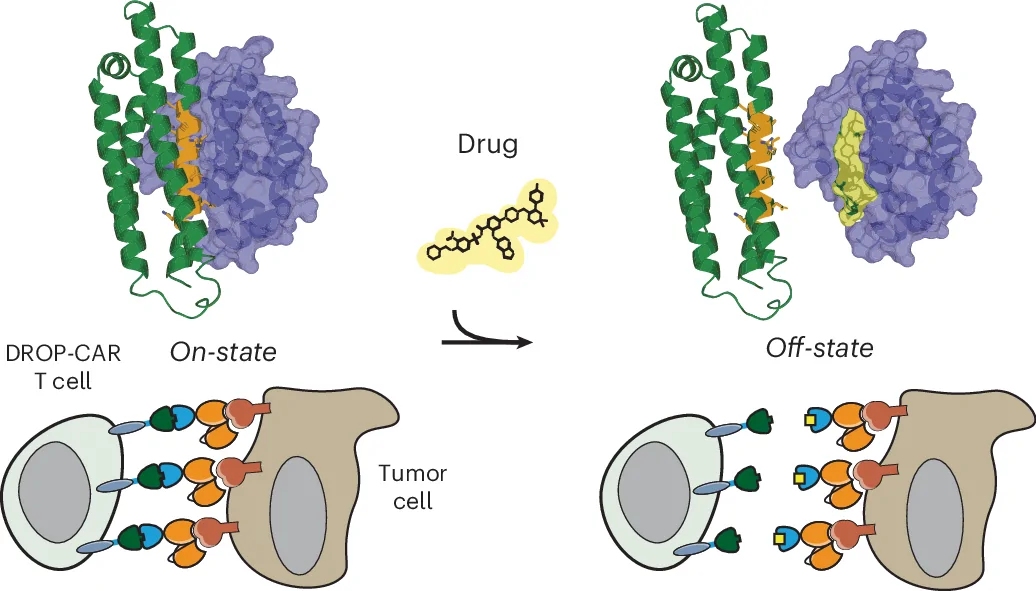

## Abstract (web summary)

Drug-controlled DROP-CARs enable reversible extracellular control of CAR T cell function via human-derived protein switches that modulate cell–cell interactions and support dual-antigen targeting as well as logic-gated signaling.

## Main

Chimeric antigen receptor (CAR) T cell therapy has achieved durable and complete responses in a high proportion of persons with certain B cell malignancies but relapse is problematic^1^. Moreover, extending such outcomes to nonhematological solid tumors, which represent the majority of cancers^2^, remains a grand challenge. Indeed, on-target, off-tumor toxicity of CAR T cells directed against solid tumor antigens, which are also present on healthy tissues with few exceptions, is a priori a safety concern^3^. In addition, as more T cell coengineering strategies and combinatorial treatments are implemented to address immunosuppressive barriers in the tumor microenvironment, the risk and incidence of toxicity from CAR therapy will likely increase^4^.

Most CARs currently used in the clinic are second-generation (2G) and comprise an antigen-binding moiety (typically a single-chain variable fragment, scFv), fused to a hinge, a transmembrane region and the endodomains of one costimulatory receptor and of CD3ζ^5^. Limitations to the 2G-CAR include that (1) chronic antigen exposure^6^ can render the engineered T cells exhausted; (2) toxicity can result from on-target reactivity against healthy tissues; and (3) overresponsiveness at high antigen density or tumor burden can trigger adverse events such as cytokine release syndrome (CRS)^7^. A variety of approaches have been developed to improve the safety of 2G-CAR T cells. For example, suicide-switches have been introduced where, once activated, they eliminate the T cells^8^. Alternatively, weaker-affinity scFvs have been used to render the 2G-CARs nonresponsive to low tumor antigen density as may be found on healthy tissue but this may also limit efficacy because of heterogeneous expression levels on tumor cells^9–12^.

On-switch and off-switch CAR designs that allow remote control of T cell activity levels by small-molecule administration represent a promising strategy for balancing function and safety^5,13^. The first on-switch CAR separated antigen binding and intracellular signaling on two distinct chains, which heterodimerized through an FK506-binding protein (FKBP) domain and a T2089L mutant of the FKBP rapamycin-binding domain in response to the rapamycin analog AP21967 (ref. ^14^). More recent efforts have shifted toward on-switch or off-switch CARs responsive to nonimmunosuppressive small molecules with longer half-lives. Examples include on-switches based on viral protease inhibition in self-cleaving CARs by the antiviral agent grazoprevir^15,16^, inducible CAR degradation by lenalidomide responsive zinc-finger degrons^17,18^ and CARs built with antibody fragments engineered such that tumor antigen binding is conditional upon the presence of a small molecule such as methotrexate^19,20^.

While there has been remarkable progress in the field, switch designs based on human-derived domains (that is, to minimize immunogenicity) that are responsive to clinically approved small molecules are rare and existing ones have limitations. For example, CAR T cell persistence and expansion correlate with response but rapamycin inhibits antigen-induced proliferation of T cells^1,21^. In addition, the immunomodulatory effects of lenalidomide, including changes in cytokine production, T cell activation and natural killer cell function^22^ can influence response and undesirable side-effects such as neutropenia, fatigue and cardiac disorders^23^ that impact tolerance can occur. Therefore, the development of novel switch designs controlled by alternative clinically approved small molecules is warranted.

Here, through rational protein design and library screening, we generated a stable protein–protein interaction (PPI) of human origin that can be efficiently disrupted by the clinically approved molecule venetoclax. We incorporated the components into a drug-regulated off-switch PPI (DROP)-CAR design including a transmembrane signaling (S)-chain that noncovalently engages through the PPI with a receptor (R)-domain. Taken together, our study demonstrates that disruption of the PPI in this configuration abrogates cell–cell contact, enabling robust and precise control over DROP-CAR T cell activity both in vitro and in vivo, and supports strong translational potential of the strategy.

## Results

### DROP-CAR design and cell-surface expression

Previously, we developed the STOP-CAR containing transmembrane S-chains and R-chains designed for reversible dissociation upon application of the small molecule A-1155463 (ref. ^24^). Briefly, the computationally designed PPI joining the chains included truncated Bcl-xL and lead design protein 3 (LD3), a human protein scaffold (apolipoprotein E4) engrafted with critical binding residues from the BH3 domain of Bim to generate a high-affinity interface. Important limitations to the design are that A-1155463 is not clinically approved, the PPI (Bcl-xL:LD3) is located in the endodomain and PPI disruption does not disengage the CAR T cell from its target.

Along with Bcl-xL, LD3 also binds to the structurally similar antiapoptotic protein Bcl-2 (*K*_D_ = 0.8 nM) and LD3:Bcl-2 can be disrupted, albeit inefficiently, by the clinically approved and orally available small molecule venetoclax^24^, a Bcl-2 inhibitor used in the treatment of various hematological malignancies^25^. Here, we sought to optimize the LD3:Bcl-2 interface for maintaining stability of the PPI but allowing efficient disruption by venetoclax. These components would then be used in a DROP-CAR design comprising an extracellular PPI enabling drug-induced receptor disruption and breakage of T cell contact with the target tumor cell.

**Fig. 1:**
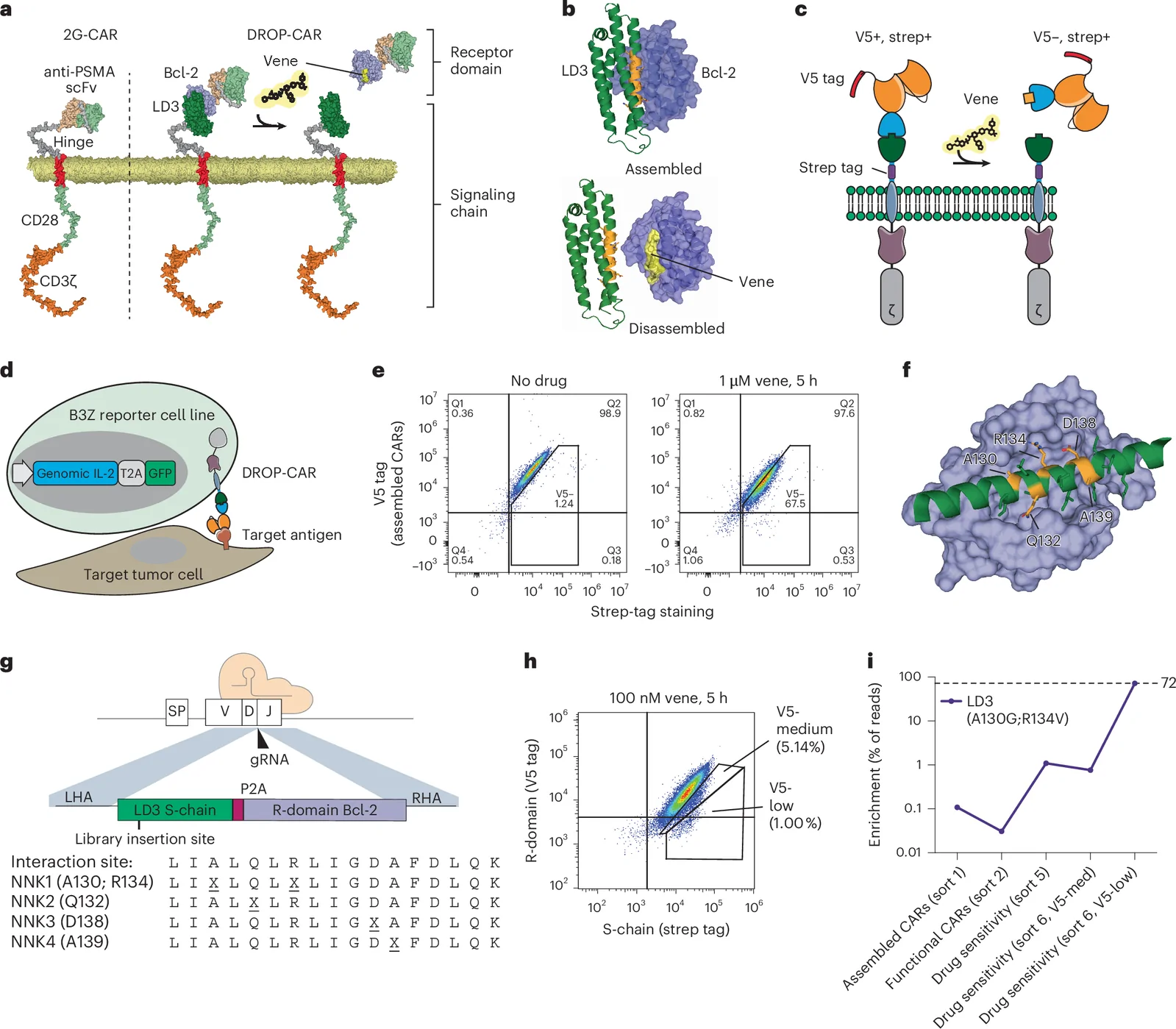
Schematic of the DROP-CAR, library design and screening to generate optimized LD3 variants. **a**, Left, a 2G-CAR consisting of the scFv (peach and mint green), hinge domain (gray), transmembrane domain (red), CD28 endodomain (light green) and CD3ζ endodomain (orange). Right, Heterodimeric DROP-CAR comprising an S-chain that includes LD3 and an R-domain made up of the tumor-targeted scFv fused to Bcl-2. In the presence of venetoclax, PPI is disrupted and the R-domain is released. **b**, Top, model of assembled LD3:Bcl-2 complex. Bottom, dissociation of LD3:Bcl-2 complex in response to venetoclax. The models are based on PDB 6IWB (LD3), PDB 6O0K (Bcl-2 and venetoclax) and AlphaFold2 models of R-domains. **c**, Illustration of tagged S-chain and R-domain and DROP-CAR disassembly upon venetoclax administration. **d**, Schematic of DROP-CAR expression in the engineered B3Z reporter cell line. **e**, Cell-surface staining of B3Z cells expressing the nonoptimized DROP-CAR comprising wild-type LD3. The V5 tag was used to stain the soluble scFv–Bcl-2 and the Strep tag was used to stain the membrane-bound LD3. Left, tag staining in the absence of venetoclax. Right, tag staining after 5 h of coincubation with 1 μM venetoclax. **f**, Major interaction site between Bcl-2 (cyan) and LD3 (green). Residues chosen for mutagenesis are highlighted in orange and labeled. The model is based on PDB 6IWB; only the major interacting helix of LD3 is shown. **g**, Site saturation mutagenesis library for three interface LD3 single mutants and one double mutant. The codons for residues marked by X were replaced with the NNK degenerate codon. **h**, Sorting for medium and low levels of V5 tag labeling (from sort 6). **i**, Enrichment of the LD3-A130G;R134V variant after indicated sorts as analyzed by deep sequencing. For sort 6, both V5-medium and V5-low were analyzed. Vene, venetoclax. Source data

We began by building a construct (Supplementary Tables 1 and 2) encoding a DROP-CAR prototype with murine components targeting HER2 and using Bcl-2:LD3 as a disruptable PPI. In comparison to a 2G-CAR that links tumor antigen binding to T cell activation in a single chain (Fig. 1a, left), the DROP-CAR is a split design including an S-chain that noncovalently associates with a nontransmembrane R-domain (Fig. 1a, right), which can be released in the presence of venetoclax (Fig. 1b). For cell-surface detection of the two DROP-CAR components, we fused a Strep tag to the hinge of the S-chain and a V5 tag to the R-domain (Fig. 1c).

The DROP-CAR construct was inserted into a Cas9-expressing B3Z reporter cell line in the T cell receptor β-chain locus by homology-directed repair (HDR)^12^ (Fig. 1d and Supplementary Table 3). Cell-surface expression of the assembled murine DROP-CAR was validated by dual-tag labeling and flow cytometric analysis and we observed limited disruption of the DROP-CAR after 5 h of incubation with 1 µM venetoclax (Fig. 1e).

### Screening for optimal DROP-CAR stability and disruption

Having demonstrated proof of principle for successful cell-surface assembly of the DROP-CAR, as well as the ability to release the R-domain upon incubation with venetoclax, we next set out to improve the PPI. In brief, we sought to augment LD3:Bcl-2 disruption by venetoclax while maintaining stability of the S-chain:R-domain complex in its native state (that is, the assembled DROP-CAR in the absence of venetoclax).

We used the Rosetta modeling suite to calculate changes in Gibbs free energy (ΔΔ*G*) from in silico site saturation mutagenesis of major LD3 interface residues (A130–A139, based on PDB 6IWB; Fig. 1f). Bcl-2 was left untouched so as not to interfere with venetoclax binding. For experimental characterization, we selected five LD3 amino acids displaying a range in ΔΔ*G*: (1) Q132 and A139 as mild targets (mean change in ΔΔ*G* = 0.1 each); (2) D138 as a highly disruptive target (mean change in ΔΔ*G* = 6.8); and (3) a combination of A130 and R134, both medium-range targets (median change in ΔΔ*G* = 3.4 and 1.3, respectively), thereby generating a total of 460 variants, including R134A and D138A previously associated with increased drug sensitivity^26^ (Supplementary Fig. 1). These variants were directly integrated into the corresponding genomic region of LD3 within the anti-HER2 DROP-CAR B3Z cells by Cas9-mediated HDR (Fig. 1g and Supplementary Fig. 2a).

In total, we performed six rounds of screening (Supplementary Figs. 2b–d) and sequence analysis was performed at key stages to track enrichment of functional and venetoclax sensitive LD3 variants (Methods). Consistent with our in silico analysis, functional CARs enriched in the library screening after sort 3 largely consisted of variants of the two sites predicted to be least disruptive to the LD3:Bcl-2 complex, Q132X and A139X. Few variants were identified for the position predicted to be most disruptive, D138X. A wide range of variants were enriched for the double mutant A130X;R134X, from undetectable to 1.4% (Supplementary Table 4). Cells with decreased R-domain staining relative to the S-chain (indicating release of the R-domain upon venetoclax treatment) were sorted (Fig. 1h and Supplementary Fig. 2e). In the final screening (sort 6) one double variant, A130G;R134V dominated, making up just over 70% of the most drug-sensitive population (V5-low). In earlier sorts, this variant was present at 0.11% (sort 1: assembled CARs), 0.03% (sort 3: functional CARs), 1.1% (sort 5: second drug-sensitivity sort) and 0.76% (sort 6: third drug-sensitivity sort, V5-medium) of the population (Fig. 1i, Supplementary Fig. 2d and Supplementary Table 4). The strong enrichment of the A130G;R134V variant of LD3 after the three drug-sensitivity screens (in sort 6, third drug-sensitivity sort, V5-low, but not in V5-medium) suggests that the optimal interface enabling both DROP-CAR stability and drug sensitivity of the PPI occupies a narrow sequence space.

### DROP-CAR selection and LD3 variant affinity measurements

We next generated DROP-CAR B3Z cells for the three most enriched variants from the third drug-sensitivity selection (sort 6, V5-low; Supplementary Fig. 2c), the LD3 variant A130G;R134V, which we named double-mutant (dm)LD3, and single mutants Q132L and A139R, and evaluated venetoclax-induced receptor disruption (Supplementary Fig. 2d). We observed minor disruption by venetoclax of DROP-CARs including wild-type LD3, partial disruption for both the Q132L and the A139R variants and major disruption for dmLD3 (Supplementary Fig. 2e). The superiority of dmLD3 is consistent with its high enrichment during the library screening. As previously mentioned, LD3 is made up of a human scaffold protein (apolipoprotein E4) computationally engrafted with ten critical binding residues of the BH3 domain of Bim^24^.Hence, a dmLD3 consists of a human protein having a total of 12 amino acid differences from the wild type (Supplementary Fig. 2f).

Lastly, we determined the *K*_D_ of dmLD3 versus the single-mutant LD3 (smLD3) variant A139R, with Bcl-2. Recombinant proteins were produced, purified and evaluated for binding affinity by surface plasmon resonance (SPR). We calculated a *K*_D_ of 82 nM for dmLD3:Bcl-2 and a *K*_D_ of 67 nM for smLD3:Bcl-2 (Supplementary Fig. 3a,b). As expected, both bound weaker than wild-type LD3:Bcl-2 (*K*_D_ of 0.8 nM), which is poorly disrupted by venetoclax^26^.

### DROP-CAR cell-surface expression and drug-induced disruption

**Fig. 2:**
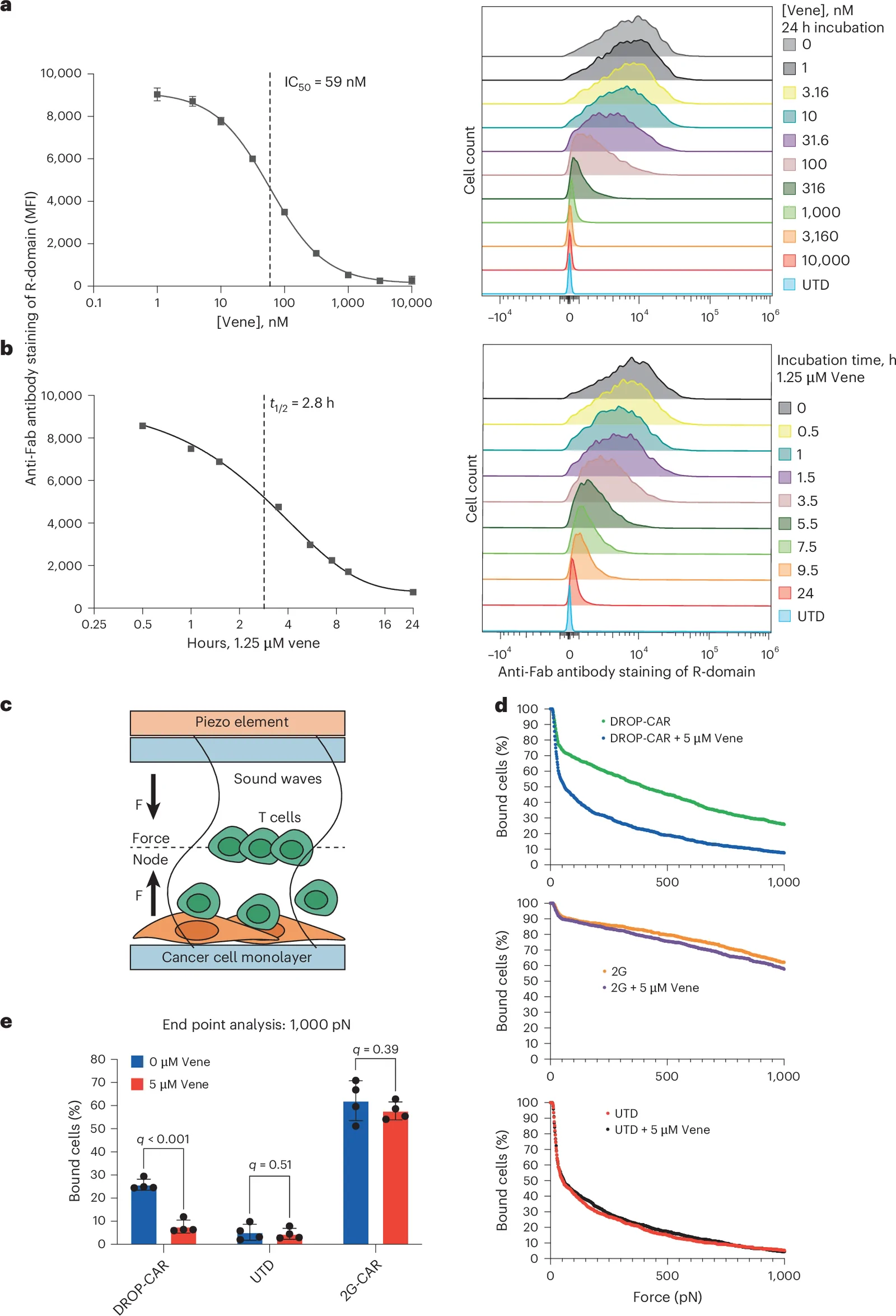
Characterization of the optimized DROP-CAR. **a**, Dose–response curve of optimized DROP-CAR (containing dmLD3) engineered Jurkat cells in response to venetoclax (IC_50_ = 59 nM). **b**, Time-course experiment of optimized DROP-CAR Jurkat cells at indicated time points after incubation with 1.25 µM venetoclax (*t*_1/2_ = 2.8 h). Transduction efficiency for the DROP-CAR was 100%. **c**, Schematic of LUMICKS experiment. **d**,**e** Percentage of DROP-CAR, 2G-CAR and UTD Jurkat cells that remained bound after 2.5 min of a linear acoustic force ramp from 0 to 1,000 pN with or without venetoclax. Partially detached Jurkat cells (hinge cells) were counted as detached. In **a**,**b**, values on the left are the mean ± s.d. of *n* = 3 biological replicates. The histograms on the right are examples for a single replicate. In **d**, values are the mean ± s.d. of *n* = 4 measurements on separate chips. The *q* values were obtained from *t*-tests with *P* values adjusted to account for multiple testing. Transduction efficiency was 59% for the DROP-CAR and 99% for the 2G-CAR. Source data

Next, we built a prostate-specific membrane antigen (PSMA)-targeted DROP-CAR using the J591 scFv^27^, human signaling components (CD28 and CD3ζ endodomains) and the optimized dmLD3:Bcl-2 off-switch (Supplementary Fig. 3c) and tested it for cell-surface expression and disruption by venetoclax. We began by engineering Jurkat cells and demonstrated near-total disassembly of the DROP-CAR after 24 h of incubation with 1 µM venetoclax, yielding a half-maximal inhibitory concentration (IC_50_) of 59 nM (95% confidence interval: 54–64 nM; Fig. 2a). In contrast, we observed no impact of venetoclax on the expression of an equivalent 2G-CAR (Supplementary Fig. 3d,e). Our findings are in range with the standard oral dose of 400 mg venetoclax given, which typically achieves a plasma concentration of 2 µM within 6 h^28,29^. We further investigated the speed of DROP-CAR disruption. With a venetoclax concentration of 1.25 µM, we observed a receptor disassembly half-life of approximately 2.8 h (95% confidence interval: 2.6–3.1 h; Fig. 2b). Given that venetoclax has a terminal half-life of approximately 19–26 h in humans^28,29^, this response rate suggests compatibility with a treatment regimen of low-dose venetoclax that can be adjusted to balance DROP-CAR T cell function and safety.

### Cell interaction testing of drug-treated DROP-CAR T cells

Having demonstrated venetoclax-mediated disruption of the DROP-CAR expressed on Jurkat cells, we next sought to test our hypothesis that the release of the R-domain would abrogate cell–cell contact. To that end, we measured the interaction strength between anti-PSMA DROP-CAR Jurkat cells and target PC3-PIP tumor cells with or without venetoclax using the LUMICKS z-Movi device (Fig. 2c). For the assay, engineered Jurkat cells and controls were incubated overnight with or without venetoclax and then added onto a monolayer of target tumor cells. After 5 min to bind, detachment was tracked by microscopy over 2.5 min while applying a force ramp from 0 to 1,000 pN. We observed a venetoclax-induced reduction in the number of bound DROP-CAR Jurkat cells but not of 2G-CAR Jurkat cells (Fig. 2d). At 1,000 pN, with partially detached cells counted as dissociated, both untransduced (UTD) and DROP-CAR Jurkat cells were less than 10% bound in the presence of venetoclax, while 2G-CAR Jurkat cells remained 60% bound. DROP-CAR Jurkat cells remained about 30% bound without venetoclax (Fig. 2e). When partially detached cells were counted as bound, at 1,000 pN in the presence of venetoclax, the UTD cells were approximately 25% bound, DROP-CAR Jurkat cells were approximately 30% bound and 2G-CAR Jurkat cells just under 90% bound. In the absence of venetoclax, DROP-CAR Jurkat cells remained 64% bound (Supplementary Fig. 4).

The comparable percentage of bound cells observed between UTD Jurkat cells and DROP-CAR Jurkat cells in the presence of venetoclax indicates that cell–cell interactions were reduced to near-background levels. This finding suggests that the disruptive forces fall within the functionally relevant range needed to discriminate antigen-specific CAR binding from nonspecific interactions, consistent with results from previous experiments^30^. The results are also consistent with the transduction efficiency for this experiment (DROP-CAR: 59%, 2G-CAR: 99%) and confirm that cell–cell interactions of DROP-CAR Jurkat T cells and target tumor cells can be regulated through venetoclax-mediated R-domain detachment from the S-chain.

### Generation of independently controlled dual DROP-CAR T cells

**Fig. 3:**
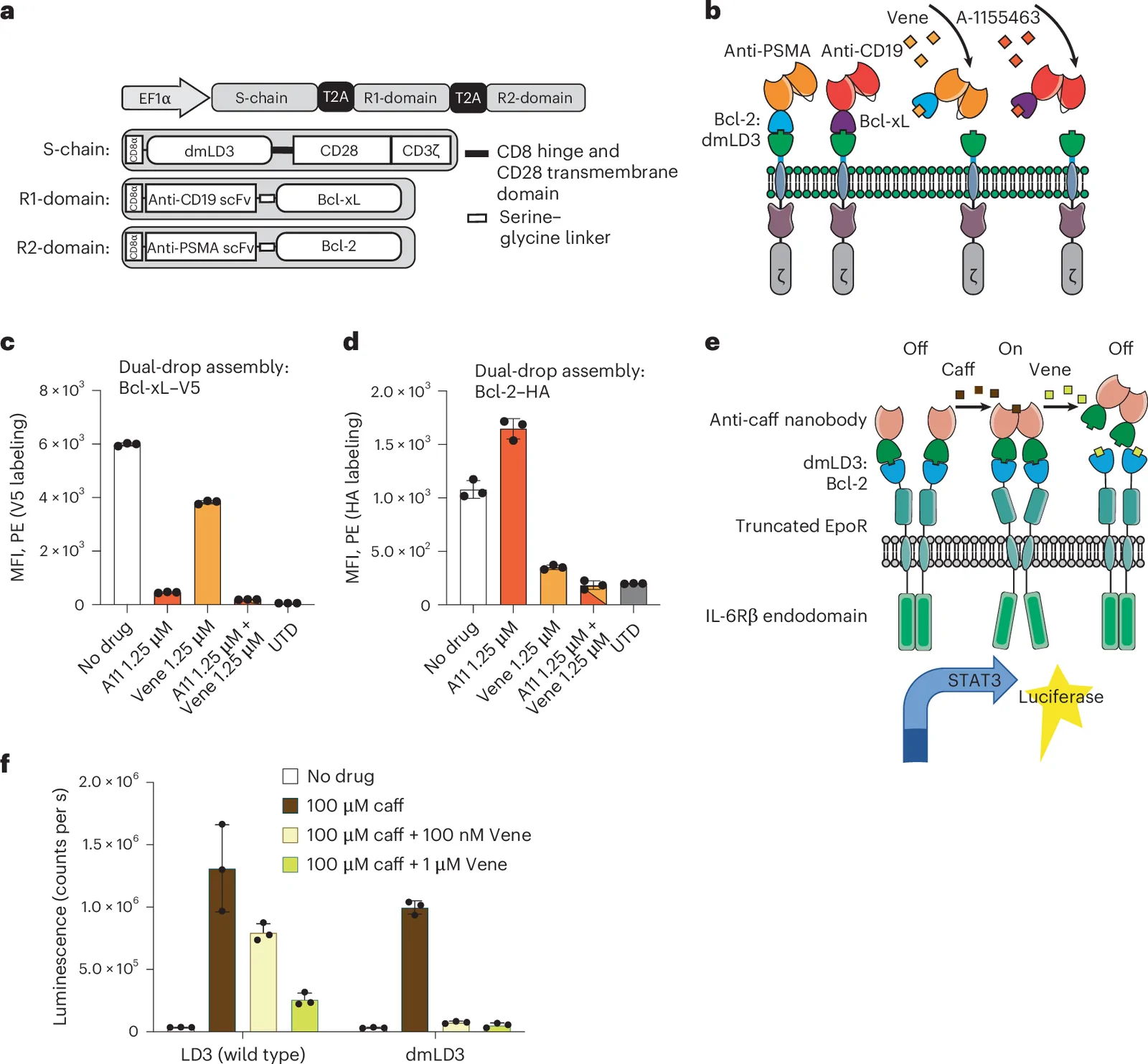
Proof of concept for dual DROP-CARs and application of the optimized switch to logic-gated receptors. **a**, Schematic of a lentiviral vector encoding the dual DROP-CARs, with one R-chain containing Bcl-xL (R1-domain) and the other one Bcl-2 (R2-domain). **b**, Schematic of cell-surface expression of two DROP-CARs, one regulated by venetoclax and the other regulated by A-1155463. **c**, Evaluation of DROP-CAR disassembly upon incubation with one or both small molecules and anti-V5 tag labeling to detect the anti-CD19 R-domain containing Bcl-xL. **d**, Testing of DROP-CAR disassembly upon incubation with one or both small molecules and anti-HA tag labeling to detect the anti-PSMA R-domain containing Bcl-2. UTD cells were used as controls to determine background labeling. **e**, A AND (NOT B) logic for caffeine and venetoclax in the GEMS cytokine receptor platform. Schematic of engineered cytokine receptors that switch-on STAT3 signaling and STAT3-induced luciferase expression in response to caffeine and switch-off in response to venetoclax. **f**, Reporter gene expression in HEK293T cells to test for A AND (NOT B) logic behavior of the system. In **c**,**d**,**f**, values are the mean ± s.d. of *n* = 3 biological replicates. Source data

A variety of strategies, including dual CARs and tandem CARs that engage more than one antigen^31^ and adaptor or universal CARs for transiently targeting antigens of choice^32,33^, have been developed to overcome antigen escape, which is an important barrier to therapy^5^. Hence, we next sought to test proof of concept for DROP-CARs targeting two different antigens. Briefly, we built a lentiviral construct encoding the S-chain containing dmLD3 followed by two different R-domains. The first R-domain targeted the B cell lineage antigen CD19 (FMC63 scFv)^34^ and was fused to Bcl-xL (responsive to A-1155463) (ref. ^24^). The second one targeted PSMA (J591 scFv)^27^ and was fused to Bcl-2 (responsive to venetoclax) (Fig. 3a,b). We tagged Bcl-xL with V5 and Bcl-2 with HA to allow us to track the disruption or loss of each R-domain upon small-molecule administration. We observed that A-1155463 led to almost complete release of Bcl-xL while venetoclax caused only partial release (Fig. 3c), consistent with its moderate inhibition of Bcl-xL (*K*_i_ = 48 µM)^35^, whereas venetoclax strongly released Bcl-2 but A-1155463 almost not at all (Fig. 3d). This demonstrates that it is feasible to differentially modulate the expression of two different DROP-CARs.

### Extending the DROP mechanism to other synthetic receptors

We next sought to evaluate whether the DROP mechanism could be extended to other synthetic receptor types and, thus, built it into the generalized extracellular molecule sensor (GEMS) cytokine receptor platform and tested it in HEK293T cells. Briefly, GEMS consist of the extracellular domain of the erythropoietin receptor (EpoR) fused to the intracellular domain of IL-6Rβ. To enable inducible dimerization, domains that respond to specific stimuli such as nanobodies that dimerize in response to caffeine are fused to the N terminus of the EpoR domain^36,37^.

We introduced the dmLD3:Bcl-2 module between the EpoR and caffeine nanobodies with the aim of creating a system that turns on in response to caffeine through dimerization of the nanobodies and turns off in response to venetoclax by causing nanobody release (Fig. 3e). The system worked with the intended behavior and we observed enhanced shutoff for dmLD3 versus wild-type LD3 (Fig. 3f). This confirms that dmLD3 forms complexes that are stable enough to yield functional synthetic cytokine receptors as well as CARs and can also be disrupted more efficiently than LD3. We subsequently confirmed that Bcl-xL and A-1155463 are also functional in this context (Supplementary Fig. 5), showing that the DROP mechanism can be extended to other synthetic receptor platforms and could be useful for building systems with elaborate logics. Notably, in a synthetic biology context, the type of on/off logic of DROP-CARs or DROP-GEMS in response to two inputs can be referred to as A AND (NOT B) or A NIMPLY B^38^. Such mechanisms may be useful to turn on receptor activity or gene expression in response to environmental cues or inducer molecules but only if a second condition is not present.

### Function of DROP-CARs versus 2G-CARs in primary human T cells

**Fig. 4:**
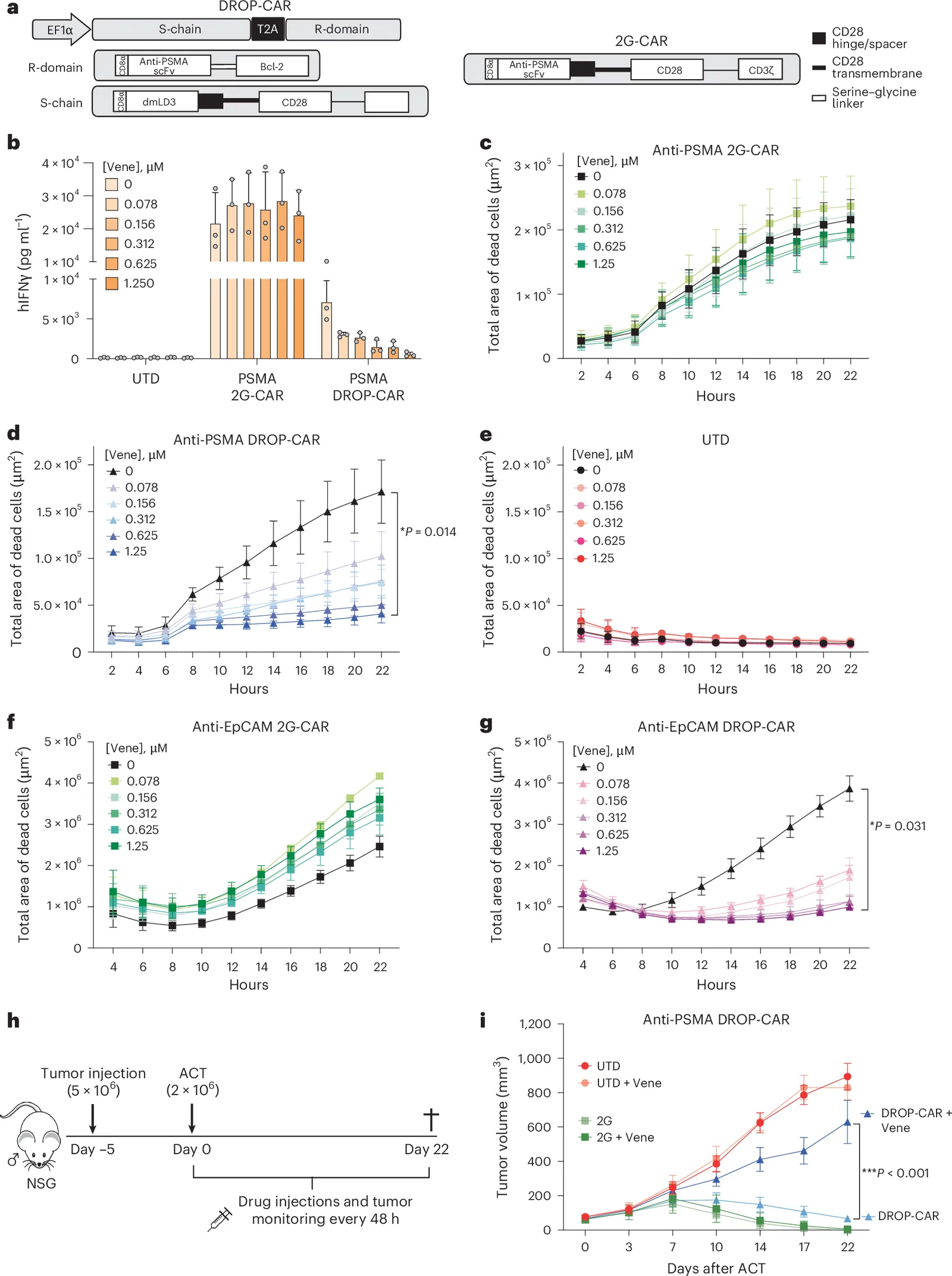
In vitro and in vivo characterization of human DROP-CAR T cells. **a**, Schematic of anti-PSMA DROP (left) and 2G (right) CAR molecules. **b**, IFNγ production by anti-PSMA CAR T cells upon 24 h of coculture with PSMA^+^ PC3-PIP cells with or without venetoclax. **c**, IncuCyte cytotoxicity assay at an effector-to-target ratio of 2:1 to evaluate PC3-PIP tumor cell killing by anti-PSMA 2G-CAR T cells. **d**,**e**, DROP-CAR T cells (**d**) or UTD T cells (**e**) in the presence of up to 1.25 μM venetoclax. **f**,**g**, IncuCyte cytotoxicity assay to evaluate (EpCAM^+^) PC3-PIP tumor cell killing by anti-EpCAM 2G-CAR T cells (**f**) or DROP-CAR T cells (**g**) in the presence of up to 1.25 μM venetoclax. In **b**,**f**,**g**, values are the mean ± s.e.m. of *n* = 3 human donors. In **c**–**e**, values are the mean ± s.e.m. of *n* = 8 human donors. **h**, Schematic of the in vivo adoptive T cell transfer study (*n* = 7 mice per group). **i**, Tumor control curves over 22 days for the adoptive T cell transfer (ACT) study. Before all functional assays, the engineered T cells were rested and adjusted by mixing them with UTD cells for equivalent transgene expression levels. A mix of 50% CD4^+^ T cells and 50% CD8^+^ T cells was used in all functional experiments (*n* = 1 HD shown; one replicate from three independent experiments is reported). Values are reported for *n* = 7 mice per group (mean ± s.e.m.). A two-way ANOVA comparing all groups was performed to assess statistical significance in **d**,**g**,**i**. Source data

Next, we sought to compare the DROP-CAR versus an equivalent 2G-CAR expressed in primary human T cells^39^ (Fig. 4a). For the DROP-CAR, we achieved 40–60% lentiviral transduction efficiency in CD4^+^ T cells and 20–40% in CD8^+^ T cells versus about 80% transduction efficiency for the 2G-CAR in both CD4^+^ and CD8^+^ T cells (Supplementary Fig. 6a). These values are in line with the general observation that transduction efficiency decreases with the size and complexity of the CAR^31,40^. Notably, these values are well above typical thresholds for clinical trials, which can be set below 10% as transduced cells are expected to expand in the presence of antigen^41^. A higher mean fluorescence intensity (MFI) was observed for the 2G-CAR, indicating that it is expressed at higher levels per cell than the DROP-CAR (Supplementary Fig. 6b). We further evaluated memory phenotype and expansion at 11 days after transduction but noted no significant differences amongst the CAR and UTD T cells (Supplementary Fig. 6c–e).

In terms of in vitro function, both 2G-CAR and DROP-CAR T cells produced interferon-γ (IFNγ) upon coculture with target PSMA^+^ PC3-PIP tumor cells (Fig. 4b), albeit at lower levels for the latter (at 24 h), corresponding to lower cell-surface expression levels of the DROP-CAR (Supplementary Fig. 6b). Importantly, coculture in the presence of 1.25 µM venetoclax abrogated IFNγ production by DROP-CAR T cells but not by 2G-CAR T cells (Fig. 4b). We subsequently set up IncuCyte real-time cytotoxicity assays and demonstrated robust and equivalent killing of PC3-PIP tumor cells by 2G-CAR and DROP-CAR T cells and a venetoclax dose-dependent decrease in cytotoxicity by DROP-CAR but not 2G-CAR T cells (Fig. 4c,d). Coculture with UTD T cells with or without venetoclax had no impact on the target tumor cells (Fig. 4e).

Previously, we demonstrated with the LUMICKS z-Movi device that venetoclax administration abrogates contacts between DROP-CAR Jurkat cells and target tumor cells (Fig. 2). To further investigate this mechanism, we performed high-resolution spinning-disk confocal live imaging of primary human DROP-CAR T cells cocultured with target tumor cells (Supplementary Fig. 7). In untreated conditions, we observed that DROP-CAR T cells formed stable and prolonged contacts with tumor cells accompanied by robust calcium influx and polarization of the microtubule-organizing center (MTOC) toward the center of the synapse (Supplementary Fig. 7a), hallmarks of productive T cell activation. However, venetoclax administration (5 μM, added 1 h before imaging) modulated DROP-CAR T cell contact with target tumor cells (Supplementary Fig. 7b) and was disruptive to target cell killing (Supplementary Fig. 7c), calcium flux (Supplementary Fig. 7d) and contact time (Supplementary Fig. 7e). Furthermore, in the presence of venetoclax, the architecture of the immune synapse was perturbed; synaptic interphases were significantly shorter (Supplementary Fig. 7f) and MTOC polarization was frequently asymmetric (Supplementary Fig. 7g), indicating compromised cytoskeletal coordination, potentially as a consequence of hampered receptor activation.

In our study, we hypothesized that a DROP-CAR design (Fig. 1a) would be superior to our original dual transmembrane STOP-CAR for which the disruptive small molecule dissociates the R-chain from the S-chain to block function in the presence of target antigen. Hence, for comparative purposes we used the optimized dmLD3:Bcl-2 switch in the STOP-CAR design, in both the extracellular (EXT-STOP) and the intracellular (INT-STOP) regions of the chains (Supplementary Fig. 8a). However, although we were able to express both the EXT-STOP-CARs and the INT-STOP-CARs on the surface of primary human T cells (Supplementary Fig. 8b) and the cells exhibited equivalent fold expansion to UTD, 2G-CAR and DROP-CAR T cells (Supplementary Fig. 8c), target cell killing by the EXT-STOP-CAR and INT-STOP-CAR T cells could not be blocked by venetoclax (Supplementary Fig. 8d). Similarly, it was not possible to abrogate IFNγ production by the EXT-STOP-CAR and INT-STOP-CAR T cells by venetoclax (Supplementary Fig. 8e).

Lastly, to test whether the DROP-CAR design could be extended to additional scFvs, we built lentiviral vectors for a 2G-CAR and a DROP-CAR targeting epithelial cellular adhesion molecule (EpCAM) with scFv C21542, also containing a CD8α hinge, the transmembrane domain and endodomain derived from CD28 and the endodomain of CD3ζ. We achieved similar T cell expansion and transduction efficiencies to equivalent anti-PSMA CARs (Supplementary Fig. 6e,f) and the CAR T cells were able to robustly kill PC3-PIP cells, which are naturally EpCAM^+^, with only the DROP-CAR T cells showing sensitivity to venetoclax (Fig. 4f,g and Supplementary Fig. 6g).

### In vivo testing of human DROP-CAR versus 2G-CAR T cells

Encouraged by the high cytolytic capacity of DROP-CAR T cells and their robust inactivation in the presence of venetoclax, we next sought to compare subcutaneous PC3-PIP tumor control in NSG mice by anti-PSMA 2G-CAR versus DROP-CAR T cells with or without venetoclax (Fig. 4h). We observed that both 2G-CAR and DROP-CAR T cells can efficiently control PC3-PIP tumors and that venetoclax administration (2.5 mg kg^−1^ every 48 h) abrogated control by the DROP-CAR- but not 2G-CAR T cells (Fig. 4i). Importantly, we showed that the suppression is reversible as, upon venetoclax withdrawal, DROP-CAR T cells regained tumor control, even at an advanced stage (Supplementary Fig. 9a). In an independent in vivo study, we observed that 2.5 mg kg^−1^ venetoclax did not fully abrogate DROP-CAR T cell activity but increasing the dose to 5 mg kg^−1^ led to tumor escape (Supplementary Fig. 9b). Upon venetoclax withdrawal at day 42, tumors regressed, indicating persistence and functional recovery of the DROP-CAR T cells (Supplementary Fig. 9c). Together, these data demonstrate remote and reversible control of DROP-CAR T cells by venetoclax in vivo.

### Comparing DROP-CAR-based and degron-based off-switch CAR T cells

**Fig. 5:**
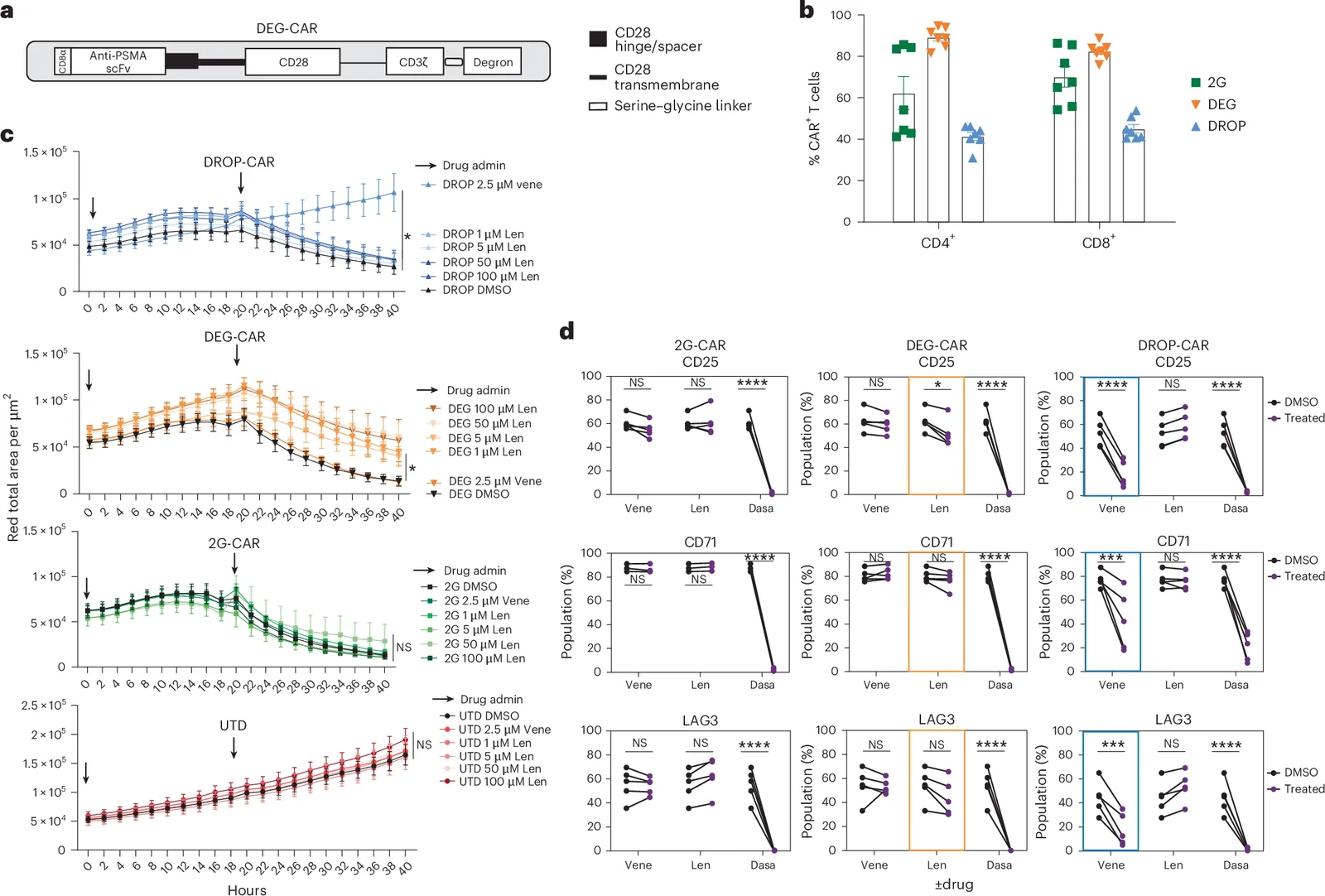
In vitro control of DROP-CAR versus DEG-CAR T cells. **a**, Schematic of anti-PSMA CAR fused to DEG-CAR responsive to lenalidomide. **b**, Transduction efficiency of 2G-CAR, DEG-CAR and DROP-CAR T cells on day 12 after transduction (*n* = 7 HDs; two independent replicates). **c**, IncuCyte cytotoxicity assay at an effector-to-target ratio of 2:1 to evaluate PC3-PIP tumor cell killing by anti-PSMA 2G-CAR, DROP-CAR and DEG-CAR T cells in the presence of 2.5 μM venetoclax (legend in bold) or 100 nM–1 μM lenalidomide (*n* = 3 HDs; two independent experiments). A two-way ANOVA was used to determine statistical significance by comparing DMSO versus small molecule (lenalidomide and/or venetoclax). **d**, Cell-surface expression of CD25, CD71 and LAG3 by the different CAR T cells upon 35 h of coculture with target tumor cells (1:1 effector-to-target ratio) with or without indicated small molecules. Data are shown as the mean ± s.e.m. (*n* = 5; two independent replicates). DEG-CAR CD25 DMSO versus lenalidomide, *P = 0.0209; DROP-CAR CD71 DMSO versus venetoclax, ***P = 0.0004; DROP-CAR LAG3 DMSO versus venetoclax, ***P = 0.0002; DROP-CAR C25 versus venetoclax, ****P < 0.0001. A two-way ANOVA was used to determine statistical significance by comparing DMSO versus treated conditions. Len, lenalidomide. Source data

A variety of different off-switch CARs have now been described^5,15,42,43^, including the use of degron tags (DEG-CARs)^17,18^ that, upon the administration of lenalidomide or its derivatives, target the receptor for ubiquitination and proteasomal degradation^44^. Given the clinical relevancy of DEG-CARs (human-derived components and a clinically approved drug), we next sought to compare our DROP-CAR to this strategy. The two approaches function through unrelated mechanisms and may, therefore, be combined in certain settings^42^. We selected a library-derived superdegron (made up of amino acids 130–189 of IKZF3 with amino acids 400–410 of ZFP91 in place of amino acids 146–156 of IKZF3) developed in ref. ^17^, fused it to the anti-PSMA 2G-CAR (Fig. 5a) and achieved cell-surface expression in primary human T cells (Fig. 5b). In target-cell-killing assays we demonstrated that 2.5 μM venetoclax prevented DROP-CAR T cells from controlling tumor cells and 100 nM–1 μM lenalidomide decreased target cell killing by DEG-CAR T cells (Fig. 5c). In contrast, neither small molecule had any impact on tumor cell killing by 2G-CAR T cells and venetoclax did not impair DEG-CAR T cells nor did lenalidomide modulate DROP-CAR T cell function (Fig. 5c).

We further assessed the expression of activation markers CD25 (IL-2 receptor α-chain) and CD71 (transferrin receptor) and immune checkpoint receptor LAG3 by T cells engineered with 2G-CAR, DEG-CAR or DROP-CAR upon PSMA^+^ target tumor cell coculture with or without the respective small molecule (Fig. 5d). As a positive control, we used the tyrosine kinase inhibitor dasatinib, which strongly blocks Lck and several other signaling molecules involved in T cell activation^45^. As expected, dasatinib fully abrogated CD25, CD71 and LAG3 upregulation by cocultured 2G-CAR, DROP-CAR and DEG-CAR T cells. We also observed significantly lower levels of these markers by DROP-CAR T cells in the presence of venetoclax and a trend for downregulation of these markers by DEG-CAR T cells treated with lenalidomide.

**Fig. 6:**
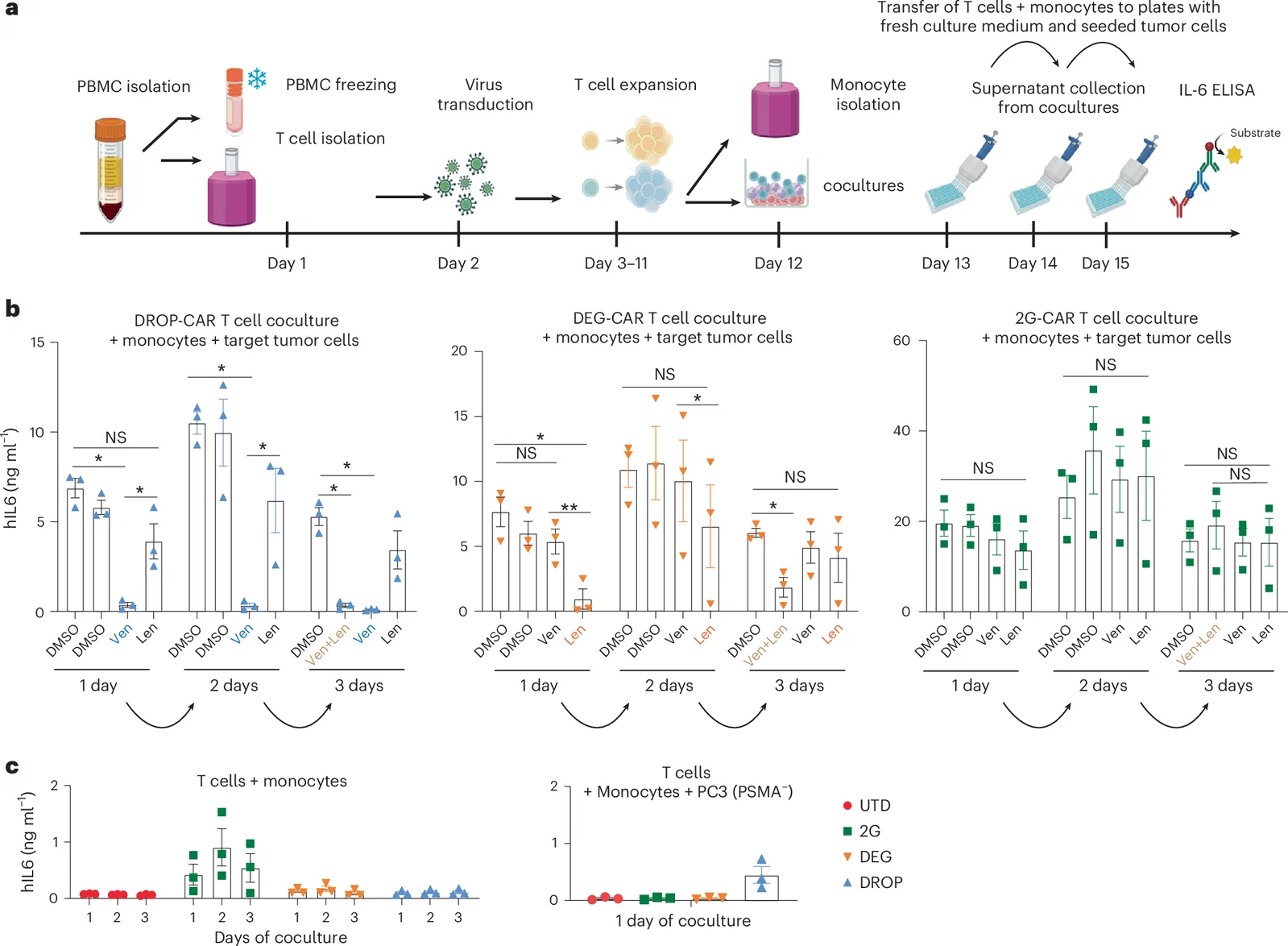
CRS potential of DROP-CAR versus DEG-CAR T cells. **a**, Schematic of the CRS potential assay, generated with BioRender.com. **b**, CRS potential assay by quantification of IL-6 production upon coculture of UTD, 2G-CAR, DEG-CAR and DROP-CAR T cells on day 12 after transduction with autologous monocytes and target tumor cells. Autologous monocytes were isolated (and donor T cell matched) on the assay day. Cocultures were established in the presence of 2.5 μM venetoclax and/or 1 μM lenalidomide (*n* = 3 HDs; two independent experiments). A two-way ANOVA was used to determine statistical significance by comparing all groups within each day. Day 1, DROP-CAR DMSO versus venetoclax, *P = 0.0113 and 0.0161. Day 2, DROP-CAR DMSO versus venetoclax *P = 0.0133; DROP-CAR DMSO versus lenalidomide *P = 0.028. Day 3, DROP-CAR DMSO versus venetoclax, *P = 0.0216; DROP-CAR DMSO versus lenalidomide + venetoclax, *P = 0.0146. Day 1, DEG-CAR DMSO versus venetoclax, *P = 0.0350; DEG-CAR DMSO versus lenalidomide, *P = 0.0464, **P = 0.0061. Day 2, DEG-CAR venetoclax versus lenalidomide, **P = 0.0043. Day 3, DEG-CAR DMSO versus lenalidomide + venetoclax, *P = 0.0711. NS, not significant. **c**, Control cultures of T cells and monocytes or T cells, monocytes and nontarget tumor cells (*n* = 3 HDs; two independent experiments). In **a**–**c**, data are reported as the mean ± s.e.m. Before all functional assays, the engineered T cells were rested and adjusted by mixing them with UTD cells for equivalent percentage CAR expression levels. A mix of 50% CD4^+^ T cells and 50% CD8^+^ T cells was used in the cocultures. Source data

Lastly, to evaluate the potential of venetoclax to mitigate CRS induced by DROP-CAR T cells, we set up an in vitro assay (schematic in Fig. 6a) in which CAR T cells are cocultured with monocytes and target tumor cells with or without the respective small molecules and IL-6 is measured in the supernatant^46^. Briefly, on day 12, the cocultures were set up and, after 24 h, the T cells and monocytes were transferred to plates seeded with fresh tumor cells and culture medium. This was repeated two more times (on days 13 and 14). Supernatants were collected on days 13, 14 and 15. We set up such side-by-side conditions for 2G-CAR, DEG-CAR and DROP-CAR T cells (Fig. 6b), as well as controls including T cells and monocytes or T cells, monocytes and nontarget tumor cells (Fig. 6c). All other control culture conditions can be found in the Methods. We observed that, in the serial cocultures, venetoclax fully abrogated IL-6 production in the context of the DROP-CAR T cells on days 1, 2 and 3 (Fig. 6b, left), whereas, for the DEG-CAR T cells, lenalidomide blocked IL-6 production in the first coculture but not in the second and third serial cocultures (Fig. 6b, middle). In contrast neither small molecule had any impact on IL-6 production in the 2G-CAR T cell cocultures (Fig. 6b, right). Hence, our in vitro data indicate the potential for venetoclax administration to mitigate CRS if triggered by DROP-CAR T cells in vivo.

## Discussion

Using structure-guided design and mammalian cell library screening, we developed an optimized off-switch composed of human protein components that can be efficiently disrupted by venetoclax. At the outset of the study, we envisioned a DROP-CAR architecture consisting of a single transmembrane chain encoding intracellular signaling modules and a releasable subunit containing the tumor-targeted scFv. We hypothesized that venetoclax-mediated disruption of the DROP-CAR design would reversibly suppress T cell reactivity by mitigating cell–cell contact. The DROP-CAR is conceptually similar to adaptor-based universal CARs^32,33^, except that the adaptor is produced by the engineered T cells and can be dissociated on demand. In contrast, traditional adaptor CARs can target multiple different tumor antigens^33^ but require repeated injections at various doses, which may increase immunogenicity risks and face challenges of tumor penetration, persistence and efficient engagement of engineered T cells^5^.

For the PPI, we began with truncated Bcl-2, which is structurally similar to Bcl-xL and also binds to our computationally designed protein LD3 with high affinity^26^. However, LD3 could not be effectively dissociated from Bcl-2 by venetoclax. Hence, we built a mammalian cell-based library of DROP-CARs with amino acid replacements at sites in LD3 predicted to be minimally to highly disruptive to Bcl-2 binding. The screening was performed in a logical manner; we first selected for cells expressing both the S-chain and the R-domain at their surface, then selected for cells expressing a functional DROP-CAR and finally enriched cells for stable DROP-CAR variants that were also sensitive to disruption by venetoclax.

Of the nearly 500 LD3 variants built into the library, only one was highly enriched, dmLD3, which maintained PPI stability while increasing disruption by venetoclax. Another variant, smLD3, emerged with a similar binding affinity to Bcl-2 (smLD3: 67 nM, dmLD3: 82 nM) but was less sensitive to venetoclax. This suggests that additional features of dmLD3, such as potentially increased flexibility or altered hydrophobicity of a potential drug entry point, may contribute to more sensitive switching. In total, dmLD3 includes the engraftment of 12 aa onto the human scaffold protein ApoE. We anticipate that the risk of immunogenicity of the DROP-switch is lower than for switches of nonhuman origin.

We demonstrated the modularity of the off-switch by combining it with caffeine-induced engineered cytokine receptors based on the GEMS platform^36,37^. Such a response pattern to two small molecules (caffeine and venetoclax) serves as proof of concept for AND/NOT logic, a fundamental logic gate for circuit design in synthetic biology^38^. Moreover, it shows that the DROP mechanism can be used to add additional layers of control to other receptor systems beyond CARs, likely including inducible zinc-finger transcription factors^16^, SynNotch^47^ and other synthetic receptors^48,49^. Additionally, we showed that the off-switch can be extended to display more than one scFv. Dual-CAR and tandem receptor designs recognizing two tumor antigens are promising for mitigating antigen escape^50^ but reliable cancer antigens are rare and simultaneous targeting of multiple markers may exacerbate on-target, off-tumor toxicities. scFv-specific control using dual DROP-CARs could, therefore, help to mitigate these risks.

We successfully engineered primary human T cells to express DROP-CARs targeting PSMA and EpCAM, demonstrating strong in vitro activity against tumor cells that was abrogated by venetoclax. The S-chain and R-domain of the DROP-CAR presumably assemble in the endoplasmic reticulum and remain stably expressed at the T cell surface until replaced through normal protein turnover. Previous studies estimated the half-life of conventional CARs at the cell surface to be approximately 6 h^51^. Using antibody staining targeting the antitumor scFv, we detected fully assembled DROP-CARs at the cell surface and demonstrated venetoclax-mediated disruption with a half-life of just under 3 h.

Although DROP-CARs were expressed at lower levels per cell than equivalent 2G-CARs, they exhibited similar cytotoxicity in IncuCyte assays. Importantly, DROP-CAR but not 2G-CAR T cell function was blocked by venetoclax. Moreover, anti-PSMA DROP-CAR T cells robustly controlled PC3-PIP tumors in vivo and their activity could be remotely and reversibly modulated upon venetoclax administration and withdrawal. A side-by-side in vitro comparison revealed more effective inhibition of DROP-CAR T cells by venetoclax than DEG-CAR T cells by lenalidomide, likely reflecting direct receptor disruption rather than reliance on slower degradation pathways. Interaction strength measurements and live-cell imaging confirmed that venetoclax disrupts the DROP-CAR, thereby regulating T cell–tumor cell contact.

Lenalidomide and venetoclax have broadly similar side-effects, although individual patient responses may vary, highlighting the value of alternative therapeutic strategies. In vitro data show that the optimized DROP-CAR was disrupted at concentrations well below typical plasma levels, with half-maximal receptor disruption occurring around 60 nM. By contrast, plasma levels after standard venetoclax dosing reach approximately 2 µM within 6 h^28,29^, suggesting that intermittent or lower-dose regimens could balance the efficacy, safety and attenuation of T cell exhaustion.

A potential limitation of the DROP-CAR is its reduced cell-surface expression compared to traditional 2G-CARs. Although lentiviral transduction efficiencies were satisfactory (40–60% in CD4^+^ T cells and 20–40% in CD8^+^ T cells), the higher MFI observed for the 2G-CAR indicates a lower surface density of the DROP-CAR, potentially because of increased size, lower integration copy number or partial misfolding. While lower CAR surface density can be advantageous in certain settings^52^ such as a high tumor burden, it may limit efficacy against tumors expressing low antigen levels.

CRS is the primary toxicity associated with CAR T cell therapy and remains an important safety issue for all commercially available CARs^7^. It is driven by CAR T cell interactions with endogenous monocytes and macrophages, hyperactivation of immune cells and cytokine release at supraphysiologic levels^53^. Using an in vitro serial coculture assay^46^, we found that venetoclax abrogated IL-6 production in the DROP-CAR T cell cocultures. In contrast, lenalidomide suppressed IL-6 production in the initial DEG-CAR T cell coculture but not upon repeated tumor cell exposure, potentially suggesting saturation of the intracellular degradation pathways.

In summary, we developed an optimized off-switch derived from human proteins and incorporated it into a DROP-CAR design, enabling reversible control of engineered primary human T cells. We further demonstrated proof of principle for dual off-switch DROP-CARs and a synthetic logic-gated cytokine receptor. The DROP-CAR system expands the toolbox of switchable CAR technologies, providing a modular framework for drug-controlled regulation of cell–cell interactions and supporting the development of advanced multi-input control strategies for cellular immunotherapies.

## Methods

### In silico LD3 interface site saturation mutagenesis

The Rosetta modeling suite was used to compute the ΔΔ*G* for the LD3:Bcl-2 complex (PDB 6IWB) upon substituting each of the ten major interface residues to all possible 20 amino acids. The structure was relaxed with the ‘FastRelax’ mover to calculate the initial ΔΔ*G*. Each interface residue of interest was substituted by side-chain repacking and energy minimization using ‘PackRotamersMover’ and ‘MinMover’, respectively. The difference in ΔΔ*G* between the mutant and initial state was then calculated. According to our computational workflow, Δ*G* refers to the change in Gibbs free energy upon protein folding, while ΔΔ*G* refers to the change upon protein complex formation, both expressed in Rosetta energy units. We compare the ΔΔ*G* values resulting from in silico site saturation mutagenesis to the ΔΔ*G* value from the wild-type complex (change in ΔΔ*G* = ΔΔ*G*_mutant_ − ΔΔ*G*_wild type_). A positive change in ΔΔ*G* indicates a weakened protein interface, predicting a reduction in the binding affinity of the mutant complex compared to the wild type. We repeated the same method three times to calculate the mean and s.d. of the results. On the basis of the results and the structure, we did not substitute L131 and L135 as they do not point toward the interface and might contribute to stabilizing the LD3 core. We discarded G137 as almost every mutant resulted in clashes with Bcl-2, likely prohibiting binding. From the remaining sites, we experimentally tested two mildly disruptive mutants (Q132 and A139), one strongly disruptive mutant (D138) and two mutants that showed a wide spread of ΔΔ*G* (A130 and R134). We tested A130 and R134 substitutions in combination to maximize the tested range of different affinities.

### Compound preparation and storage

Venetoclax (>99.9%; Chemietek, CT-A199) and A-1155463 (99.5%; Chemietek, CT-A115) were prepared in DMSO (Neofroxx, 1264) as 10 mM stocks and aliquots were stored at −20 °C. Caffeine was prepared in Milli-Q water as 100 mM stocks and aliquots were stored at −20 °C. Lenalidomide (SML2283-250MG, Merk) was prepared in DMSO as 1 M stocks and aliquots were stored at −20 °C. Dasatinib (S1021-100MG, LubioScience) was resuspended in DMSO at 20 mM stocks and aliquots were stored at −20 °C.

### Protein structure visualizations

The models in Figs. 1 and 2a were generated with PyMol^40^. Figure 1a was assembled from AlphaFold2 (ref. ^54^) models of individual R-domains for visualization purposes and is not an accurate portrayal of a real protein structure.

### Cell culture

The prostate carcinoma cell line PC3-PIP and Jurkat T cell line were cultured in RPMI-1640 (Gibco, 72400047). B3Z cells were cultured in IMDM (Gibco, 31980030). HEK293T cells were cultured in DMEM (Gibco, 10566016) for experiments and RPMI-1640 for virus production. SKOV3 cells were cultured in DMEM/F-12 (Gibco, 31331028). All media were supplemented with heat-inactivated 10% FBS (Gibco, 26140079) and penicillin–streptomycin (Gibco, 10378016). All cells were cultured at 37 °C and 5% CO_2_ in a humidified incubator.

### HEK293T cell experiments and reporter assay

For the HEK293T cell experiments and reporter gene assay (in Fig. 4), 16,000 cells per well were seeded with 100 µl of complete DMEM in the inner 60 wells of a 96-well plate, 24 h before transfection. The DNA mix for 12 wells consisted of 600 ng of receptor plasmid (S193), 600 ng of secreted LD3–nanobody fusion (pLS147 or pLS912), 200 ng of STAT3 expression plasmid (pLS392), 300 ng of reporter plasmid for STAT3-dependent nanoluciferase secretion (mixed with 660 µl of DMEM without additives) and 8,250 ng of polyethyleneimine (PEI; Polysciences, 24765-1). All plasmids are described in Supplementary Table 1. The transfection mixture was vortexed and incubated for 20 min; then 50 µl per well was added to the cells. These numbers correspond to ~130 ng of DNA and 625 ng of PEI per well. After 16 h, the medium with the transfection mix was replaced with 100 µl per well of fresh complete medium containing the indicated drugs. After 2 h, supernatant samples were collected for quantification of the secreted reporter protein nanoluciferase. For the assay, 5 µl of cell supernatant was mixed with 5 µl of a Nano-Glo substrate:buffer solution (Promega, N1110) at a 1:50 ratio in a black 384-well plate and analyzed using a multiplate reader. A more detailed protocol is available from a previous study^37^.

### Protein expression and purification

Both dmLD3 and smLD3 with a C-terminal His_6_ tag were produced using the Expi293TM expression system (Thermo Fisher Scientific, A14635). First, 6 days after transfection, the supernatant was isolated and filtered, while the protein was purified using Ni-NTA affinity columns on an ÄKTA pure system (GE healthcare), followed by size-exclusion chromatography in PBS. The purified proteins were then concentrated, aliquoted and stored at −80 °C for subsequent experiments. The Bcl-2 protein for SPR experiments was produced previously^55^.

### SPR assay for protein–protein binding affinities

To determine the binding affinities between the LD3 variants and Bcl-2 proteins, SPR measurements were conducted using a Biacore 8K (GE Healthcare). The Bcl-2 protein was immobilized on a CM5 chip (GE Life Science) at 5 µg ml^−1^ for 140 s of contact time in pH 4.5 sodium acetate solutions. Serial dilutions of the mutant LD3 proteins were prepared in HBS-EP buffer (10 mM HEPES, 150 mM NaCl, 3 mM EDTA and 0.005% surfactant P20; GE Life Science) and flown over the immobilized chips. The binding affinities (*K*_D_) were determined using a steady-state binding model or equilibrium model with the Biacore 8K evaluation software.

### Plasmid construction

All plasmids are described in Supplementary Table 1 and all relevant protein sequences are listed in Supplementary Table 2. DNA for cloning was ordered from Twist Bioscience, either as gene fragments or as readily cloned vectors. The scFv–bcl-xL insert in the dual DROP-CAR was purchased from GenScript. Gene fragments were ordered with flanking regions overlapping with the target vector and inserted by Gibson assembly. The initial DROP-CAR DNA was cloned into a pUC57 vector containing the homology arms for HDR in the B3Z cells. The DROP-CARs for Jurkat and primary T cell experiments were cloned into a third-generation self-inactivating lentiviral expression vector, pELNS, with expression driven by the EF1α promoter. Recombinant LD3 variants were expressed from a pHLSec vector with a strong CAG promoter. GEMS and secreted LD3–nanobody fusion proteins were expressed from plasmids with a weak SV40 promoter.

### Lentivirus production

First, 24 h before transfection, 10 million HEK-293 T cells were seeded in 16.5 ml of complete DMEM in a T-150 tissue culture flask. Plasmid DNA was purified using the Endo-free maxiprep kit (Qiagen, 12362). For transfection, a mixture of 7 µg of pVSV-G (VSV glycoprotein expression plasmid), 18 µg of R874 (Rev and Gag/Pol expression plasmid) and 15 µg of pELNS-based vector was combined with 180 µl of Turbofect (Thermo Fisher Scientific, R0533) and 3 ml of Opti-MEM (Gibco, 31985062). After 48 h of incubation, the viral supernatant was collected and viral particles were concentrated by ultracentrifugation for 2 h at 24,000*g*, before being resuspended in 400 µl of complete RPMI-1640 medium and snap-frozen on dry ice.

### Jurkat cell transduction

Jurkat cells were seeded at a density of 1 million cells per ml in 48-well plates with 500 µl per well. For each transduction, 50 µl of viral supernatant was added to the wells. Following 24 h of incubation at 37 °C, the cell medium was replaced and the cells were incubated for an additional 72 h at 37 °C.

### Primary human T cell transduction and culture

Primary human T cells were extracted from the peripheral blood mononuclear cells (PBMCs) of healthy donors (HDs). PBMCs were separated using Lymphoprep (Axonlab, 12053231) and CD4^+^ and CD8^+^ T cells were isolated using a magnetic bead-based negative selection kit (easySEP; StemCell Technologies, 17951) according to the manufacturer’s instructions. The isolated T cells were cultured in complete RPMI and stimulated with Dynabeads human T activator CD3/CD28 for T cell expansion and activation (Gibco, 11132D) at a 1:2 ratio of T cells to beads. T cells were transduced (0.25 million CD4^+^ and CD8^+^ cells for in vitro experiments and 3 million cells for in vivo experiments) with lentiviral particles 18–22 h after activation. Human recombinant IL-2 (PeproTech, 200-02) was added every other day at a concentration of 50 IU per ml until day 5 after stimulation. On day 5, the beads were removed and IL-2 was replaced with IL-7 and IL-15 (Miltenyi Biotec, 130-095-367 and 130-096-567) at 10 ng ml^−1^ each. The cells were maintained at a density of 0.5–1 million cells per ml for expansion until day 11 or 12. Before functional assays, engineered T cells were rested and mixed with UTD cells to standardize transgene expression levels. Unless stated otherwise, a mix of 50% CD4^+^ T cells and 50% CD8^+^ T cells was used in all functional experiments.

### Cytokine production assays

For cytokine release assays, an effector-to-target ratio of 1:1 was used. Briefly, 50,000 T cells per well were cocultured with an equal number of target cells in 96-well round-bottom plates in a final volume of 200 µl of complete RPMI medium. After 24 h, supernatant samples were collected to measure IFNγ, IL-2 and IL-6 using commercial ELISA kits according to the manufacturer’s instructions (BioLegend, 430801, 431801 and 430515).

### Cytotoxicity assays

Cytotoxicity assays were conducted using an IncuCyte Instrument at a 2:1 effector-to-target ratio (50% CD4^+^ and 50% CD8^+^ T cells) in complete RPMI medium containing Cytotox red reagent (final concentration: 125 nM; Essen Bioscience, 4632) without exogenous cytokines. Target cells (12,500) were seeded in flat-bottom 96-well plates and, after 4 h of incubation, 25,000 washed and rested T cells (no cytokine addition for 48 h) were added per well in a final volume of 200 µl. Images were taken every 2 h and the total red area per well was calculated using IncuCyte ZOOM software. Cytotoxicity is reported as the total area of dead cells as measured by Cytotox red reagent uptake (red area per µm^2^). Background fluorescence at time 0 was subtracted from all subsequent time points. Data are presented as the mean of total red area for different HD cells ± s.e.m.

### Control of IL-6 release in coculture assays

The assay was set up (schematic in Fig. 5e) and performed according to the method described by Nouri et al.^46^. Briefly, PSMA^+^ PC3-PIP target tumor cells were seeded in a 96-well round-bottom plate at 10^4^ cells per well (coculture on first day), at 0.5 × 10^4^ cells per well (coculture on second day) and 0.25 × 10^4^ cells per well (coculture on third day). T cells were counted, normalized for percentage of CAR expression and then resuspended at 0.4 × 10^6^ cells per ml (2 × 10^4^ in 100 µl). Monocytes were isolated from autologous PBMCs on the day of the start of the coculture assay using the EasySep human monocyte isolation kit (StemCell Technologies) and resuspended at 10^5^ cells per ml. Coculture was then set up in a 2:1:1 ratio of T cells, monocytes and tumor cells with or without small molecule (venetoclax or lenalidomide). Negative controls included cultures of (1) T cells, monocytes and nontarget tumor cells (PSMA^−^ PC3 tumor cells); (2) UTD and CAR T cells alone; (iii) monocytes alone; (iv) T cells and tumor cells; or (5) T cells and monocytes. Supernatant was collected at 24 h from the first day of coculture and, at the same time, the T cells and monocytes were transferred to wells on a second plate with fresh tumor cells and fresh medium. This was repeated two more times, resulting in three supernatant collection points. Human IL-6 was then measured in the medium collected from the coculture over the 3 days by ELISA (Biolegend, 430515; human IL-6 detection kit).

### Mice and in vivo experiments

NSG mice were bred and housed in a pathogen-free facility at the Epalinges Campus of the University of Lausanne. All experiments followed Swiss Federal Veterinary Office guidelines and were approved by the Cantonal Veterinary Office. Five mice were housed per cage and provided with an enriched environment and unrestricted access to food and water. Mice were monitored at least every 2 days for signs of distress and were killed using carbon dioxide overdose at the endpoint. To evaluate tumor control by DROP-CAR T cells, 8–12-week-old NSG males were subcutaneously injected with 5 million PC3-PIP tumor cells. Groups of 7–9 mice (as indicated in the figure legends) were formed once tumors became palpable (day 5) to ensure similar mean tumor volume and s.d. The mice were treated with a peritumoral injection of 2 million T cells (UTD, 2G-CAR or DROP-CAR T cells). Then, 2 h after T cell transfer, the mice received peritumoral injections of 2.5 mg kg^−1^ venetoclax or vehicle control (DMSO) every 2 days until the endpoint or as indicated to evaluate the regain of function of DROP-CAR T cells. Tumor volumes were measured every 2 days using the formula *V* = 1/2(length × width^2^), where length is the greatest longitudinal diameter and width is the greatest transverse diameter, determined by caliper measurement.

### Genome editing

All sequences related to genome editing are listed in Supplementary Table 3. To generate the initial DROP-CAR in stably Cas9-expressing B3Z cells, purified PCR product of the gene cassette with ~700-bp flanking homology arms left and right of the genomic cut site was used for the transfection as the template for HDR. For library generation, we used short 125-nt single-stranded oligodeoxynucleotides (ssODNs; 500 pmol of ultramer; IDT) with about 60 nt left and right as homology regions and phosphorothioate bonds at each end as the template. Electroporations were performed with the SF Lonza kit on a Lonza 4D-Nucleofector by resuspending 5 × 10^4^ B3Z cells in SF buffer in a total volume of 100 µl and running program CA-138 in nucleocuvettes, followed by the addition of 600 µl of warm complete RPMI medium. To generate the guide RNA (gRNA), we heated and assembled 2.25 µl of 200 µM CRISPR RNA (crRNA) and 2.25 µl of 200 µM *trans*-activating crRNA and transfected 4 µl of the mixture together with 5 µg of template DNA. Analysis or sorting was performed after a minimum of 4 days. We confirmed successful genomic editing by extracting genomic DNA from at least 10^4^ harvested cells using the QuickExtract protocol (Lucigen, QE09050). Correct HDR template integration was validated through Sanger sequencing of the PCR amplification of the target locus with at least one primer annealing outside the integration site.

### DROP-CAR library generation

A gRNA (Supplementary Table 3) targeting the LD3 region of the initial DROP-CAR was used to introduce a frameshift deletion that removed CAR expression. After sequencing the genomic region of single clones without CAR expression, we selected three clones with small deletions and confirmed the function of the GFP reporter from the genomic IL-2 site through PMA and ionomycin stimulation. We used four gRNAs to target the LD3 site in these mutants and repaired this deletion with 125-bp ssODNs as HDR templates containing one or two degenerate NNK codons that encode all 20 aa but only a single stop codon (Supplementary Table 3). The best clone–gRNA–ssODN combination resulted in 15% frameshift repair and 5% HER2 binding recovery.

### Flow cytometry of B3Z cells

DROP-CAR expression in B3Z cells (a mouse T cell hybridoma expressing a TCR that recognizes the OVA peptide (SIINFEKL)/H-2Kb complex) was evaluated by labeling with a 1:200 dilution of biotinylated anti-Strep tag antibody (GenScript, A01737) binding the transmembrane-domain-containing chain of the DROP-CAR, followed by a 1:400 dilution of brilliant violet 421–streptavidin conjugate (Biolegend, 405225). To select for DROP-CAR assembly, we used a 1:20 dilution of an anti-V5 antibody (Invitrogen, 12-6796-42) that binds to the soluble R-domain (that is released upon venetoclax incubation). Functional DROP-CARs were selected on the basis of inducible GFP expression from the genomic IL-2 site and HER2 binding (2.5 µg ml^−1^ soluble HER2 antigen (Merck) and subsequent 1:200 APC-labeled anti-HER2 antibody (Biolegend)).

### B3Z cell DROP-CAR library screening and sequencing

For the screening, DROP-CAR library cells were cocultured in a 1:1 ratio with 3 × 10^6^ HER2-expressing SKOV3 cells in complete IMDM for 16 h. All cells were then collected, washed and sorted for GFP positivity. Cells were allowed to recover for 3–5 days before the next selection step. A total of six rounds of screening was performed. First, we sorted the library for cell-surface expression of both the S-chain and R-domain (that is, DROP-CAR cell-surface assembly) by dual-tag labeling (sort 1). Subsequently, to remove LD3 variants generating nonfunctional DROP-CARs, we cocultured the cells from sort 1 with the HER2^+^ ovarian tumor cell line SKOV3 and selected for activated library cells through expression of a GFP reporter gene (genomically integrated downstream of exon 4 of IL-2). This was performed twice (sorts 2 and 3). We then conducted three sequential library screenings for venetoclax sensitivity (sorts 4–6). In brief, for the sensitivity screenings, upon 5 h of incubation with 100 nM venetoclax, the cells were labeled with antibodies targeting the Strep tag (on the S-chain) and the V5 tag (on the R-domain). Cells with decreased R-domain staining relative to the S-chain (indicating release of the R-domain upon venetoclax treatment) were subsequently sorted.

After indicated selection rounds, we amplified the 301-bp LD3-containing region within the CAR gene and sent it for deep sequencing (GENEWIZ). Only sequences with a complete LD3 region harboring a single-amino-acid substitution were included in the subsequent analysis. Sequence analysis was performed for (1) sort 1 to ascertain the diversity of LD3 variants that maintain Bcl-2 binding; (2) sort 3 to evaluate LD3 variants present in functional DROP-CARs; (3) sort 5 to evaluate the range of LD3 variants sensitive to venetoclax disruption; and (4) sort 6 (V5-medium and V5-low labeled cells) to compare LD3 variants having moderate and high sensitivity to venetoclax disruption.

### Dose response, time-course experiments and flow cytometry of CAR-engineered Jurkat T cells

Jurkat T cells (1 × 10^4^ cells per well) were seeded into the inner 60 wells of round-bottom 96-well plates, 24 h before analysis. For the dose response experiment and for the 24-hour time point of the time course, 50 µl of complete RPMI containing the appropriate doses of venetoclax was added immediately. In the time-course experiment, on the following day, 50 µl of complete RPMI with 3.75 µM venetoclax (yielding a final concentration of 1.25 µM) was added at the specified time points before analysis. CAR assembly was evaluated by labeling with an anti-Fab antibody (1:100; Jackson ImmunoResearch, 115-606-072), which binds to the soluble scFv portion of the receptor. The MFI shift of this population was used to quantify receptor disassembly in response to venetoclax. The V5–Bcl-xL–scFv_CD19_ domain for the dual DROP-CAR was labeled with an anti-V5 antibody (1:100; Invitrogen, 12-6796-42) to determine receptor disassembly in response to A-1155463. The HA–Bcl-2–scFv_PZ1_ domain for the dual DROP-CAR was labeled with an anti-HA antibody (1:100; Miltenyi Biotec, 130-120-791) and an anti-mouse secondary antibody (1:100; Invitrogen, 12401082).

### Cell binding force assay

PC3-PIP target cells were harvested using Accutase (PAN-Biotech, P10-21200) and seeded on poly(L-lysine)-coated (Sigma-Aldrich, P4832) z-Movi microfluidic chips (Lumicks) at 3.5 × 10^7^ cells per ml. Chips with confluent monolayers were incubated for at least 2.5 h at 37 °C before measurement of binding force on the z-Movi Analyzer (Lumicks). Viability of effector cells was assessed using a Vi-CELL BLU cell viability analyzer (Beckman Coulter). Per experimental condition, 1 × 10^6^ effector cells were labeled with 1 μM Cell Trace far-red reagent (Thermo Fisher Scientific, C34564) for 15 min at 37 °C, 5% CO_2_. Effector cells were resuspended at 1 × 10^7^ cells per ml in 1× RPMI-1640 supplemented with GlutaMAX (Thermo Fisher Scientific, 61870-010), 10% fetal calf serum (PAN-Biotech, P40-37500), 1% penicillin–streptavidin (PAN-Biotech, P06-07100) and 1 % HEPES (Sigma-Aldrich, H0887).

Binding force was measured and analyzed using Oceon software (v.1.5.4) according to manufacturer recommendations. Effector cells were serially flushed into the chips. During a coincubation time of 5 min, effector cells were allowed to form contacts with target cells. Next, a force ramp ranging from 0 to 1,000 pN was applied over 2.5 min and effector cell detachment was monitored by fluorescent imaging with the z-Movi device. Binding force was determined on a single-cell level by correlation of the detachment time point with force applied. Runs with <100 or >800 effector cells detected in the measured field of view were excluded. A maximum of eight runs were performed per chip and monolayer viability was assessed by trypan blue staining after the last run. Chips with severe viability loss or monolayer cell detachment during the course of the experiment were excluded. During the analysis, effector cells stuck on glass surface or covered by force nodes were automatically deselected and cells that were moved by force but did not entirely reach the force nodes were automatically defined as hinge cells by the Oceon software. Hinge cells were either treated as bound or as detached cells, as indicated. Automatic analysis and cell deselection were reviewed but not modified by the experimenter to enable reproducibility. In case of severe errors in the automated analysis, chips were excluded from the analysis.

### Flow cytometry of primary T cells

To evaluate anti-PSMA 2G-CAR, DEG-CAR and DROP-CAR expression on primary cells, transduced cells were stained with AlexaFluor 647-conjugated anti-mouse F(ab)′ antibody (Jackson ImmunoResearch, 115-606-072). To evaluate anti-EpCAM 2G-CAR, DEG-CAR and DROP-CAR expression on primary cells, transduced cells were stained with AlexaFluor 647-conjugated anti-human F(ab)′ antibody (LubioScience, 109-607-008). Cell viability was assessed using a near-infrared fluorescent reactive dye (APC–Cy-7; Invitrogen, Life Technologies, L34976A). For phenotypic memory analysis, the following monoclonal antibodies were used: BV711 mouse anti-human CD3 (BD Bioscience, 563725), BV605 mouse anti-human CD4 (Biolegend, 317438), APC-labeled anti-human CD8 (Biolegend, 344722), PE–Texas red-labeled mouse anti-human CD45RA (Beckman Coulter, B49193) and BV421 mouse anti-human CCR7 (Biolegend, 353207). The memory phenotype was determined by first gating the CD3^+^ population and then separating the CD4^+^ and CD8^+^ subsets. These subsets were subsequently analyzed for CD45RA and CCR7 expression to ascertain the proportions of naive, central memory, effector memory and terminally differentiated effector memory RA T cells.

### Phenotypic analysis of CAR T cells cultured with target tumor cells and small molecules

T cells were transduced and normalized for equivalent percentage of 2G-CAR, DEG-CAR and DROP-CAR expression, counted and cocultured with PSMA^+^ PC3-PIP tumor cells at an effector-to-target ratio of 1:1 in presence of DMSO, venetoclax (2 μM), lenalidomide (1 μM) or dasatinib (200 nM). At 35 h after culture, T cells were stained with surface markers CD25, CD71 and LAG3 (BioLegend) following the manufacturer’s instructions. To discriminate dead cells, fixable near-infrared dead-cell stain (Thermo Fisher Scientific) was used.

### Spinning-disk live imaging assay

Live imaging was performed as previously described^56^. Briefly, PC3-PIP tumor cells were labeled with CSFE 647 as indicated by the manufacturer (Thermo Fisher, C34572), washed twice and seeded in IBIDI 18-well µ-chamber slides (Ibidi, 81816) at a concentration of 50 000 cells per well and left to adhere for 20 min. CD4^+^ nontransduced or DROP-CAR transduced T cells were stained for F-actin and tubulin (Spirochrome, SPY555–actin and SPY555–tubulin) for 1 h as indicated by the manufacturer. Venetoclax was added at 5 μM for 1 h before imaging with the indicated conditions. CD4^+^ T cells were added to tumor cells in a 1:1 ratio. RPMI (R10) medium was used for imaging. Furthermore, 1 μM of caspase 3/7 (Sigma, SCT105) and 1 µM Fluo-4 AM (Invitrogen, F14201) dye were added to each well shortly before imaging to visualize cell death and calcium influx, respectively. The best timing for immune synapse formation and cell death was empirically determined in long-term timelapse pilot experiments. Dishes were mounted on the stage of a Nikon T2 Yokogawa CSU-W1 spinning-disk confocal microscope at 37 °C, 5% CO_2_, 88% humidity. Imaging was performed in confocal mode, with a perfect focus system, using a ×40 objective. Three *z* stacks of 3-µm distance were acquired per condition, covering a *z* space of 6 µm total. Framerates were 110 s and 18 positions per slide were imaged, with one position per well. Tumor cells only served as a control for cell death evoked by imaging conditions (<2%). Images were obtained using 555-nm (for T cells and dead cells), 647-nm (for tumor cells) and 488-nm (every fifth frame, calcium influx) laser channels, as well as in bright-field mode. The best imaging interval was determined to be between 1 and 6 h of coculture. We imaged T cells from *n* = 3 different HDs. Image analysis was performed using Fiji/ImageJ and custom macros. Immune synapse formation is defined as a stable cell–cell contact of >4 min.

### Statistics

All statistical calculations were performed using GraphPad Prism (versions 8.3–10). Binding affinities in the SPR drug competition assays were determined using three-parameter nonlinear regression with the equation *Y* = bottom + (top − bottom)/(1 + (*x*/IC_50_)). Significance for killing assays or in vivo experiments was calculated using a two-way analysis of variance (ANOVA) for multiple comparisons. Exact *P* values are shown in the figures. The representative data from cell assays are presented as individual values overlayed with bars of mean values and error bars for the s.d. or s.e.m. as indicated in the legend. For primary T cells, *n* refers to the number of human donors. For Jurkat and HEK293T cells, *n* = 3 represents biological replicates, which are defined as different wells on the same plate. IC_50_ values in cell assays were calculated using three-parameter nonlinear regression. The *t*_1/2_ values were determined using the equation *Y* = (*Y*₀ − plateau) × exp(−*k* × *x*) + plateau for one-phase decay analysis.

### Reporting summary

Further information on research design is available in the Nature Portfolio Reporting Summary linked to this article.

## Online content

Any methods, additional references, Nature Portfolio reporting summaries, source data, extended data, supplementary information, acknowledgements, peer review information; details of author contributions and competing interests; and statements of data and code availability are available at https://doi.org/10.1038/s41589-026-02152-x.

## Supplementary information

Supplementary Information Supplementary Tables 1–4 and Figs. 1–9.

Reporting Summary

## Source data

Source Data Fig. 1 Source data.

Source Data Fig. 2 Source data.

Source Data Fig. 3 Source data.

Source Data Fig. 4 Source data.

Source Data Fig. 5 Source data.

Source Data Fig. 6 Source data.

## Data Availability

All data are available in the main text or the Supplementary Information. All plasmid descriptions and protein sequences are provided in Supplementary Tables 1 and 2. Plasmids are available upon request. Source data are provided with this paper.
