# Supplementary material for: Drug-controlled CAR T cells through the regulation of cell–cell interactions
Source: Nat Chem Biol. 2026 Feb 19;22(10):1654–65. doi: 10.1038/s41589-026-02152-x (PMC13612217; doi:10.1038/s41589-026-02152-x)
Supplement: Supplementary file 1 [file 41589_2026_2152_MOESM1_ESM.pdf]

nature chemical biology

Supplementary information

https://doi.org/10.1038/s41589-026-02152-x

Drug-controlled CAR T cells through the regulation of cell–cell interactions

In the format provided by the authors and unedited

Supplementary Information

Supplementary Tables S1-S4

Supplementary Figures S1-S9

Supplementary Table 1: Description of all plasmids used in the study.

Name	Description	Figure
pVSV-G	VSV glycoprotein expression plasmid	All transductions
R874	Rev and Gag/Pol expression plasmid	All transductions
pLS400_DROP_WT	Template plasmid for making the PCR with homology arms to integrate the initial DROP CAR into B3Z cells.	4e, S2
pAM193_pHLSec_LD3_A130G-R134V	Expression plasmid (CMV enhancer with a chicken B-actin promoter) for the mutated LD3 protein A130G; R134V	S2e, S3
pAM191_pHLSec_LD3_A139R	Expression plasmid (CMV enhancer with a chicken B-actin promoter) for the mutated LD3 protein A139R	S2e, S3
pLS147_secLD3-caff	Secretion of LD3-caffeine-nanobody fusion protein for assembly on the GEMS receptors (SV40 promoter)	3, S5
pLS912_secLD3-A130G-R134V-caff	Secretion of LD3-A130G-R134V-caffeine-nanobody fusion protein for assembly on the GEMS receptors (SV40 promoter)	3, S5
pLS392_STAT3	STAT3 expression plasmid (SV40 promoter)	3, S5
pLS858_Nanoluc	STAT3 dependent nanoluciferase reporter plasmid (STAT3 binding sites – minimal CMV promoter – Nanoluciferase-Fc)	3, S5
S184-Bcl-xL-GEMS	Bcl-xL-GEMS receptor (SV40 promoter), developed by Shui et al. (2021) https://doi.org/10.1038/s41467-021-25735-9	3, S5
S193-Bcl2-GEMS	Bcl2-GEMS receptor (SV40 promoter), developed by Shui et al. (2021) https://doi.org/10.1038/s41467-021-25735-9	3, S5
pLSX135_dual-DROP-aCD19-Bcl-xL-aPSMA-Bcl-2	Lentiviral vector (EF1α Promoter – ORF – WPRE) for expressing the optimized dual-DROP-CAR	3
pLSX052_DROP-CAR_A130G-R134V	Lentiviral vector (EF1α Promoter – ORF – WPRE) for expressing the optimized PSMA-DROP-CAR	All PSMA DROP-CAR experiments unless specified differently, including all in vivo experiments.

pGGA001_pELNS-EpCAM-DROP-CAR	Lentiviral vector (EF1α Promoter – ORF – WPRE) for expressing the optimized EpCAM-DROP-CAR	4
pGGA002_INT-Split-CAR	Lentiviral vector (EF1α Promoter – ORF – WPRE) for expressing the INT-Split-CAR	S8
pGGA003_EXT-Split-CAR	Lentiviral vector (EF1α Promoter – ORF – WPRE) for expressing the EXT-Split-CAR	S8
pGGA004_Degron-CAR	Lentiviral vector (EF1α Promoter – ORF – WPRE) for expressing the Degron-CAR	5, S6

Supplementary Table 2: Protein sequences of different chimeric receptors.

Name	Description	Sequence
LD3 WT	The original LD3 protein developed in Giordano-Attianese & Gainza et al. 2020, https://doi.org/10.1038/s41587-019-0403-9	GQRWELALGRFLEYLSWVSTLSEQVQEEL LSSQVTQELRALMDETMKELKAYKSELEEQ LTPVAEETRARLSKELQAAQARLGADMED VRGRLVQYRGEVQAMLGQSTEELRVRLAS HLIALQLRLIGDAFDLQKRLAVYQAGA
LD3 A130G; R134V	The most enriched variant during sorting and the basis for the final DROP-CAR	GQRWELALGRFLEYLSWVSTLSEQVQEEL LSSQVTQELRALMDETMKELKAYKSELEEQ LTPVAEETRARLSKELQAAQARLGADMED VRGRLVQYRGEVQAMLGQSTEELRVRLAS HLI**G**LQL**V**LIGDAFDLQKRLAVYQAGA
LD3 A139R	Another partially enriched variant, not chosen for the DROP-CAR	GQRWELALGRFLEYLSWVSTLSEQVQEEL LSSQVTQELRALMDETMKELKAYKSELEEQ LTPVAEETRARLSKELQAAQARLGADMED VRGRLVQYRGEVQAMLGQSTEELRVRLAS HLIALQLRLIGD**R**FDLQKRLAVYQAGA
LD3 Q132L	Another partially enriched variant, not chosen for the DROP-CAR	GQRWELALGRFLEYLSWVSTLSEQVQEEL LSSQVTQELRALMDETMKELKAYKSELEEQ LTPVAEETRARLSKELQAAQARLGADMED VRGRLVQYRGEVQAMLGQSTEELRVRLAS HLIAL**L**LRLIGDAFDLQKRLAVYQAGA
Bcl-2	Bcl-2 sequence based on PDB 4LVT. Used in the plasmids pLS400 and S193.	AHPGRTGYDNREIVMKYIHYKLSQRGYEW DAGDDVEENRTEAPEGTESEVVHLTLRQA GDDFSRRYRRDFAEMSSQLHLTPFTARGR FATVVEELFRDGVNWGRIVAFFEFGGVMC VESVNREMSPLVDNIALWMTEYLNRHLHT WIQDNGGWDAFVELYGP
Bcl-2_{opt}	Bcl-2 sequence optimized for better solubility by Lajoie et al. 2020 https://doi.org/10.1126/science.aba6527 Used in the plasmids pLSX052, pLSX135, and pGGA001-pGGA003	AHAGRTGYDNREIVMKYIHYKLSQRGYEW DAGDDAEENRTEAPEGTESEVVHRALRDA GDDFERRYRRDFAEMSSQLHLTPDTARQR FETVVEELFRDGVNWGRIVAFFEFGGVMC VESVNREMSPLVDNIAEWMTEYLNRHLHT WIQDNGGWDAFVELYGP
Bcl-xL	Bcl-xL sequence used for dual DROP-CARs (pLSX135_dual-DROP-aCD19-Bcl-xL-aPSMA-Bcl-2) and DROP-GEMS (S184-Bcl-xL-GEMS).	MSQSNRELVVDFLSYKLSQKGYSWSQFSD VEENRTEAPEGTESEAVKQALREAGDEFEL RYRRAFSDLTSQLHITPGTAYQSFEQVVNE LFRDGVNWGRIVAFFSFGGALCVESVDKE MQVLVSRIAAWMATYLNDHLEPWIQENGG WDTFVELYGNNAAAESRKGQER
PZ1 scFv	scFv for the PZ1 DROP-CAR.	EVQLQQSGPELVKPGTSVRISCKTSGYTFT EYTIHWVKQSHGKSLEWIGNINPNNGGTTY NQKFEDKATLTVDKSSSTAYMELRSLTSED SAVYYCAAGWNFDYWGQGTTVTVSSGGG GSGGGGSGGGGSDIVMTQSHKFMSTSVG DRVSIICKASQDVGTAVDWYQQKPGQSPK LLIYWASTRHTGVPDRFTGSGSGTDFTLTIT NVQSEDLADYFCQQYNSYPLTFGAGTMLD LKR
Her2 scFv	scFv for the WT DROP-CAR for screening in BZ1 cells.	DIQMTQSPSSLSASVGDRVTITCRASQDVN TAVAWYQQKPGKAPKLLIYSASFLYSGVPS RFSGSRSGTDFTLTISSLQPEDFATYYCQQ HYTTPPTFGQGTKVEIKRTGSTSGSGKPGS

		GEGSEVQLVESGGGLVQPGGSLRLSCAASGFNIKDTYIHWVRQAPGKGLEWVARIYPTNGYTRYADSVKGRFTISADTSKNTAYLQMNSLRAEDTAVYYCSRWGGDGFYAMDVWGQGTLVTVSS
CD19 scFv	scFv used for dual DROP-CARs (pLSX135_dual-DROP-aCD19-Bcl-xL-aPSMA-Bcl-2).	DIQMTQTTSSLSASLGDRVTISCRASQDISKYLNWYQQKPDGTVKLLIYHTSRLHSGVPSRFSGSGSGTDYSLTISNLEQEDIATYFCQQGNTLPYTFGGGTKLEITGSTSGSGKPGSGEGSTKGEVKLQESGPGLVAPSQSLSVTCTVSGVSLPDYGVSWIRQPPRKGLEWLGVIWGSETTYYNSALKSRLTIIKDNSKSQVFLKMNSLQTDDTAIYYCAKHYYYGGSYAMDYWGQGTSVTVS
EpCAM scFv	scFv for the EpCAM DROP-CAR.	QVKLQQSGAELVRPGASVKLSCKASGYTFTNYWINWVKQRPGQGLEWIGNIYPSYIYTNYNQEFKDKVTLTVDESSSTAYMQLSSPTSEDSAVYYCTRSPYGYDEYGLDYWGQGTTVTVSSGGGGSGGGGSGGGGSDIELTQSPSSLTVTAGEKVTMNCKSSQSLLNSRNQKNYLTWYQQKPGQPPKLLIYWASTRESGVPDRFTGSGSGTDFTLTISSVQAEDLAVYYCQNDYVYPLTFGAGTKLEIKR
Bcl-2 GEMS	Bcl2-GEMS receptor (SV40 promoter, Plasmid: S193-Bcl2-GEMS), developed by Shui et al. (2021), used to test AND-NOT logic with co-secreted LD3-caffeine nanoboy fusions. Kappa chain secretion peptide, Bcl-2, EpoR ECD and TMD, IL6-ST ICD. Linkers uncolored.	MSETDTLLLWVLLLWVPGSTGDMAHPGRTGYDNREIVMKYIHYKLSQRGYEWDAGDDVEENRTEAPEGTESEVVHLTLRQAGDDFSRRYRRDFAEMSSQLHLTPFTARGRFATVVEELFRDGVNWGRIVAFFEFGGVMCVESVNREMSPLVDNIALWMTEYLNRHLHTWIQDNGGWDAFVELYGPTSGAPSPSLPDPKFESKAALLASRGSEELLCFTQRLEDLVCFWEEAASSGMDFNYSFSYQLEGESRKSCSLHQAPTVRGSVRFWCSLPTADTSSAVPLELQVTEASGSPRYHRIIHINEVVLLDAPAGLLARRAEEGSHVVLRWLPPPGAPMTTHIRYEVDVSAGNRAGGTQRVEVLEGRTECVLSNLRGGTRYTFAVRARMAEPSFSGFWSAWSEPASLLTASDLDPLILTLSLILVLISLLLTVLALLSAAANKRDLIKKHIWPNVPDPSKSHIAQWSPHTPPRHNFNSKDQMYSDGNFTDVSVVEIEANDKKPFPEDLKSLDLFKKEKINTEGHSSGIGGSSCMSSSRPSISSSDENESSQNTSSTVQASTVVHSGYRHQVPSVQVFSRSESTQPLLDSEERPEDLQLVDHVDGGDGILPRQQYFKQNCSQHESSPDISHFERSKQVSSVNEEDFVRLKQQISDHISQSCGSGQMKMFQEVSAADAFGPGTEGQVERFETVGMEAATDEGMPKSYLPQTVRQGGYMPQASTGV*
LD3-anti-caff fusion protein	LD3-anti-caff fusion protein (SV40 promoter, Plasmid: pLS147_secLD3-WT-caff), used to test AND-NOT logic with Bcl-2-GEMS. Albumin secretion peptide, LD3, anti-caffeine VHH (PDB 6QTL). Linkers uncolored.	MKWVTFISLLFLFSSAYSTGTSGQRWELALGRFLEYLSWVSTLSEQVQEELLSSQVTQELRALMDETMKELKAYKSELEEQLTPVAEETRARLSKELQAAQARLGADMEDVRGRLVQYRGEVQAMLGQSTEELRVRLASHLIALQLRLIGDAFDLQKRLAVYQAGAGASGSQVQLVESGGGLVQAGGSLRLSCTASGRTGTIYSMAWFRQAPGKEREFLATVGWSSGITYYMDSVKG

		RFTISRDKGKNTVYLQMDSLKPEDTAVYYC<br>TATRAYSVGYDYWGQGTQVTVSSR*
DROP_WT	WT-DROP CAR sequence before mutations. Expressed genomically from the murine BZ1 T-cell line. (template plasmid: pLS400_DROP_WT).<br>Kappa chain secretion peptide, LD3-WT, Strep tag, mCD28 hinge, mCD28 TMD, mCD28 ICD (LL:GG mutant), mCD3Z, P2A, Her2 scFv (4D5), Bcl-2, V5. Linkers uncolored.	METDTLLLWVLLLWVPGSTGDTGQRWELA<br>LGRFLEYLSWVSTLSEQVQEELLSSQVTQE<br>LRALMDETMKELKAYKSELEEQLTPVAEET<br>RARLSKELQAAQARLGADMEDVRGRLVQY<br>RGEVQAMLGQSTEELRVRLASHLIALQLRLI<br>GDAFDLQKRLAVYQAGAAESANWSHPQFE<br>KGGGGSGGGGSNWSHPQFEKSAIEFMYP<br>PPYLDNERSNGTIIHIKEKHLCHTQSSPKLF<br>WALVVVAGVLFCYGLLVTVALCVIWTNSRR<br>NRGGQSDYMNMTPRRPGLTRKPYQPYAP<br>ARDFAAYRPRAKFSRSAETAANLQDPNQL<br>YNELNLGRREEYDVLEKKRARDPEMGGKQ<br>QRRRNPQEGVYNALQKDKMAEAYSEIGTK<br>GERRRGKGHDGLYQGLSTATKDTYDALHM<br>QTLAPRGGGGSGATNFSLLKQAGDVEENP<br>GPGGGMETDTLLLWVLLLWVPGSTGDASD<br>IQMTQSPSSLSASVGDRVTITCRASQDVNT<br>AVAWYQQKPGKAPKLLIYSASFLYSGVPSR<br>FSGSRSGTDFTLTISSLQPEDFATYYCQQH<br>YTTPPTFGQGTKVEIKRTGSTSGSGKPGSG<br>EGSEVQLVESGGGLVQPGGSLRLSCAASG<br>FNIKDTYIHWVRQAPGKGLEWVARIYPTNG<br>YTRYADSVKGRFTISADTSKNTAYLQMNSL<br>RAEDTAVYYCSRWGGDGFYAMDVWGQG<br>TLVTVSSGGPGMAHPGRTGYDNREIVMKYI<br>HYKLSQRGYEWDAGDDVEENRTEAPEGT<br>ESEVVHLTLRQAGDDFSRRYRRDFAEMSS<br>QLHLTPFTARGRFATVVEELFRDGVNWGRI<br>VAFFEFGGVMCVESVNREMSPLVDNIALW<br>MTEYLNRHLHTWIQDNGGWDAFVELYGPG<br>GGSGKPIPNPLLGLDST*
PZ1-DROP-CAR	DROP-CAR expressed from the lentiviral pELNS vector (pLSX052_DROP-CAR_A130G-R134V).<br>CD8 secretion signal, FLAG, LD3 A130G; R134V, hCD8 hinge, hCD28 TMD, hCD28 ICD, hCD3Z, T2A, CD8 secretion signal, PZ1 scFv, $Bcl2_{opt}$, myc. Linkers uncolored.	MALPVTALLLPLALLLHAARPGSDYKDDDD<br>KGQRWELALGRFLEYLSWVSTLSEQVQEE<br>LLSSQVTQELRALMDETMKELKAYKSELEE<br>QLTPVAEETRARLSKELQAAQARLGADME<br>DVRGRLVQYRGEVQAMLGQSTEELRVRLA<br>SHLIGLQLVLIGDAFDLQKRLAVYQAGAAST<br>TTPAPRPPTPAPTIASQPLSLRPEACRPAA<br>GGAVHTRGLDFACDFWVLVVVGGVLACYS<br>LLVTVAFIIFWVRSKRSRLLHSDYMNMTPR<br>RPGPTRKHYQPYAPPRDFAAYRSRVKFSR<br>SADAPAYQQGQNQLYNELNLGRREEYDVL<br>DKRRGRDPEMGGKPRRKNPQEGLYNELQ<br>KDKMAEAYSEIGMKGERRRGKGHDGLYQ<br>GLSTATKDTYDALHMQALPPRSGGSGEGR<br>GSLLTCGDVEENPGPMALPVTALLLPLALLL<br>HAARPEVQLQQSGPELVKPGTSVRISCKTS<br>GYTFTEYTIHWVKQSHGKSLEWIGNINPNN<br>GGTTYNQKFEDKATLTVDKSSSTAYMELRS<br>LTSEDSAVYYCAAGWNFDYWGQGTTVTVS<br>SGGGGSGGGGSGGGGSDIVMTQSHKFMS<br>TSVGDRVSIICKASQDVGTAVDWYQQKPG<br>QSPKLLIYWASTRHTGVPDRFTGSGSGTDF<br>TLTITNVQSEDLADYFCQQYNSYPLTFGAG<br>TMLDLKRGGPGAHAGRTGYDNREIVMKYI

		HYKLSQRGYEWDAGDDAEENRTEAPEGT<br>ESEVVHRALRDAGDDFERRYRRDFAEMSS<br>QLHLTPDTARQRFETVVEELFRDGVNWGRI<br>VAFFEFGGVMCVESVNREMSPLVDNIAEW<br>MTEYLNRHLHTWIQDNGGWDAFVELYGPS<br>MREQKLISEEDL*
INT-Split-CAR	INT split-CAR expressed from the lentiviral pELNS vector (pGGA002_INT-Split-CAR).<br>CD8 secretion signal, PZ1 scFv, hCD8 hinge, hCD28 TMD, hCD28 ICD, LD3 A130G; R134V, T2A, myc, DAP10, Bcl2$_{opt}$, hCD3Z Linkers uncolored.	MALPVTALLLPLALLLHAARPVQLQQSGPE<br>LVKPGTSVRISCKTSGYTFTEYTIHWVKQS<br>HGKSLEWIGNINPNNGGTTYNQKFEDKATL<br>TVDKSSSTAYMELRSLTSEDSAVYYCAAG<br>WNFDYWGQGTTVTVSSGGGGSGGGGSG<br>GGGSDIVMTQSHKFMSTSVGDRVSIICKAS<br>QDVGTAVDWYQQKPGQSPKLLIYWASTRH<br>TGVPDRFTGSGSGTDFTLTITNVQSEDLAD<br>YFCQQYNSYPLTFGAGTMLDLKRASTTTPA<br>PRPPTPAPTIASQPLSLRPEACRPAAGGAV<br>HTRGLDFACDFWVLVVVGGVLACYSLLVTV<br>AFIIFWVRSKRSRLLHSDYMNMTPRRPGPT<br>RKHYQPYAPPRDFAAYRSHMGGGGSGGG<br>GSGGGGSGQRWELALGRFLEYLSWVSTL<br>SEQVQEELLSSQVTQELRALMDETMKELK<br>AYKSELEEQLTPVAEETRARLSKELQAAQA<br>RLGADMEDVRGRLVQYRGEVQAMLGQST<br>EELRVRLASHLIGLQLVLIGDAFDLQKRLAV<br>YQAGAAERKRRSGSGRSGSGEGRGSLLT<br>CGDVEENPGPGSMALPVTALLLPLALLLHA<br>ARPEQKLISEEDLQTTPGERSSLPAFYPGT<br>SGSCSGCGSLSLPTTTPAPRPPTPAPTIAS<br>QPLSLRPEACRPAAGGAVHTRGLDFACDP<br>RFWVLVVVGGVLACYSLLVTVAFIIFWVRSK<br>RSRLLHSDYMNMTPRRPGPTRKHYQPYAP<br>PRDFAAYRSPGGGGGSGGGGSGGGGSA<br>HAGRTGYDNREIVMKYIHYKLSQRGYEWD<br>AGDDAEENRTEAPEGTESEVVHRALRDAG<br>DDFERRYRRDFAEMSSQLHLTPDTARQRF<br>ETVVEELFRDGVNWGRIVAFFEFGGVMCV<br>ESVNREMSPLVDNIAEWMTEYLNRHLHTWI<br>QDNGGWDAFVELYGPSMRGGGGSGGGG<br>SGGGGSMHRVKFSRSADAPAYQQGQNQL<br>YNELNLGRREEYDVLDKRRGRDPEMGGKP<br>RRKNPQEGLYNELQKDKMAEAYSEIGMKG<br>ERRRGKGHDGLYQGLSTATKDTYDALHMQ<br>ALPPR*
EXT-Split-CAR	EXT split-CAR expressed from the lentiviral pELNS vector (pGGA003_EXT-Split-CAR).<br>CD8 secretion signal, PZ1 scFv, hCD8 hinge, hCD28 TMD, hCD28 ICD, hCD3Z , T2A, LD3 A130G; R134V, myc, Bcl2$_{opt}$, Linkers uncolored.	MALPVTALLLPLALLLHAARPGSDYKDDDD<br>KGQRWELALGRFLEYLSWVSTLSEQVQEE<br>LLSSQVTQELRALMDETMKELKAYKSELEE<br>QLTPVAEETRARLSKELQAAQARLGADME<br>DVRGRLVQYRGEVQAMLGQSTEELRVRLA<br>SHLIGLQLVLIGDAFDLQKRLAVYQAGAAST<br>TTPAPRPPTPAPTIASQPLSLRPEACRPAA<br>GGAVHTRGLDFACDFWVLVVVGGVLACYS<br>LLVTVAFIIFWVRSKRSRLLHSDYMNMTPR<br>RPGPTRKHYQPYAPPRDFAAYRSRVKFSR<br>SADAPAYQQGQNQLYNELNLGRREEYDVL<br>DKRRGRDPEMGGKPRRKNPQEGLYNELQ<br>KDKMAEAYSEIGMKGERRRGKGHDGLYQ<br>GLSTATKDTYDALHMQALPPRSGGSGEGR

		GSLLTCGDVEENPGPMALPVTALLLPLALLLHAARPEVQLQQSGPELVKPGTSVRISCKTSGYTFTEYTIHWVKQSHGKSLEWIGNINPNNGGTTYNQKFEDKATLTVDKSSSTAYMELRSLTSEDSAVYYCAAGWNFDYWGQGTTVTVSSGGGGSGGGGSGGGGSDIVMTQSHKFMSTSVGDRVSIICKASQDVGTAVDWYQQKPGQSPKLLIYWASTRHTGVPDRFTGSGSGTDFTLTITNVQSEDLADYFCQQYNSYPLTFGAGTMLDLKRGGPGAHAGRTGYDNREIVMKYIHYKLSQRGYEWDAGDDAEENRTEAPEGTESEVVHRALRDAGDDFERRYRRDFAEMSSQLHLTPDTARQRFETVVEELFRDGVNWGRIVAFFEFGGVMCVESVNREMSPLVDNIAEWMTEYLNRHLHTWIQDNGGWDAFVELYGPSMRTTTPAPRPPTPAPTIASQPLSLRPEACRPAAGGAVHTRGLDFACDFWVLVVVGGVLACYSLLVTVAFIIFWVRSKRSRLLHSDYMNMTPRRPGPTRKHYQPYAPPRDFAAYRS*
CD19, PZ1 Dual-DROP-CAR	Dual DROP-CAR expressed from the lentiviral pELNS vector (pLSX135_dual-DROP-aCD19-Bcl-xL-aPSMA-Bcl-2). CD8 secretion signal, FLAG, LD3 A130G; R134V, hCD8 hinge, hCD28 TMD, hCD28 ICD, hCD3Z, T2A, CD8 secretion signal, V5, CD19 scFv, Bcl-xL, T2A, CD8 secretion signal, HA, PZ1 scFv, Bcl2$_{opt}$, myc. Linkers uncolored.	MALPVTALLLPLALLLHAARPGSDYKDDDDKGQRWELALGRFLEYLSWVSTLSEQVQEELLSSQVTQELRALMDETMKELKAYKSELEEQLTPVAEETRARLSKELQAAQARLGADMEDVRGRLVQYRGEVQAMLGQSTEELRVRLASHLIGLQLVLIGDAFDLQKRLAVYQAGAASTTTPAPRPPTPAPTIASQPLSLRPEACRPAAGGAVHTRGLDFACDFWVLVVVGGVLACYSLLVTVAFIIFWVRSKRSRLLHSDYMNMTPRRPGPTRKHYQPYAPPRDFAAYRSRVKFSRSADAPAYQQGQNQLYNELNLGRREEYDVLDKRRGRDPEMGGKPRRKNPQEGLYNELQKDKMAEAYSEIGMKGERRRGKGHDGLYQGLSTATKDTYDALHMQALPPRSGGSGEGRGSLLTCGDVEENPGPMALPVTALLLPLALLLHAARPGSKPIPNPLLGLDSTGGGDIQMTQTTSSLSASLGDRVTISCRASQDISKYLNWYQQKPDGTVKLLIYHTSRLHSGVPSRFSGSGSGTDYSLTISNLEQEDIATYFCQQGNTLPYTFGGGTKLEITGSTSGSGKPGSGEGSTKGEVKLQESGPGLVAPSQSLSVTCTVSGVSLPDYGVSWIRQPPRKGLEWLGVIWGSETTYYNSALKSRLTIIKDNSKSQVFLKMNSLQTDDTAIYYCAKHYYYGGSYAMDYWGQGTSVTVSGGGGMSQSNRELVVDFLSYKLSQKGYSWSQFSDVEENRTEAPEGTESEAVKQALREAGDEFELRYRRAFSDLTSQLHITPGTAYQSFEQVVNELFRDGVNWGRIVAFFSFGGALCVESVDKEMQVLVSRIAAWMATYLNDHLEPWIQENGGWDTFVELYGNNAAAESRKGQERGRKRRSGSGEGRGSLLTCGDVEENPGPMALPVTALLLPLALLLHAARPYPYDVPDYAGGGSEVQLQQSGPELVKPGTSVRISCKTSGYTFTEYTIHWVKQSHGKSLEWIGNINPNNGGTTYNQKFEDKATLTVDKSSSTAYMELRSLTSEDSAVYYCAAGWNFDYWGQGTTVTVSSGGGGSGGGGSGGGGSDIVMTQSHKFMSTSVGDRVSIICKASQDVGTAVDWYQQKPGQSP

		KLLIYWASTRHTGVPDRFTGSGSGTDFTLTI<br>TNVQSEDLADYFCQQYNSYPLTFGAGTML<br>DLKRGGPGAHAGRTGYDNREIVMKYIHYKL<br>SQRGYEWDAGDDAEENRTEAPEGTESEV<br>VHRALRDAGDDFERRYRRDFAEMSSQLHL<br>TPDTARQRFETVVEELFRDGVNWGRIVAFF<br>EFGGVMCVESVNREMSPLVDNIAEWMTEY<br>LNRHLHTWIQDNGGWDAFVELYGPSMRE<br>QKLISEEDL*
Degron-CAR	Degron-CAR expressed from the lentiviral pELNS vector (pGGA004_Degron-CAR).<br><br>CD8 secretion signal, PZ1 scFv, hCD8 hinge, hCD28 TMD, hCD28 ICD, hCD3Z , T2A, Degron by Jan et al. 2021 https://www.science.org/doi/10.1126/scitranslmed.abb6295, Linkers uncolored.	MALPVTALLLPLALLLHAARPGSASTTTPAP<br>RPPTPAPTIASQPLSLRPEACRPVQLQQSG<br>PELVKPGTSVRISCKTSGYTFTEYTIHWVK<br>QSHGKSLEWIGNINPNNGGTTYNQKFEDK<br>ATLTVDKSSSTAYMELRSLTSEDSAVYYCA<br>AGWNFDYWGQGTTVTVSSGGGGSGGGG<br>SGGGGSDIVMTQSHKFMSTSVGDRVSIICK<br>ASQDVGTAVDWYQQKPGQSPKLLIYWAST<br>RHTGVPDRFTGSGSGTDFTLTITNVQSEDL<br>ADYFCQQYNSYPLTFGAGTMLDLKRAAGG<br>AVHTRGLDFACDFWVLVVVGGVLACYSLL<br>VTVAFIIFWVRSKRSRLLHSDYMNMTPRRP<br>GPTRKHYQPYAPPRDFAAYRSIDRVKFSRS<br>ADAPAYQQGQNQLYNELNLGRREEYDVLD<br>KRRGRDPEMGGKPRRKNPQEGLYNELQK<br>DKMAEAYSEIGMKGERRRGKGHDGLYQGL<br>STATKDTYDALHMQALPPRFNVLMVHKRS<br>HTGERPFLQCEICGFTCRQKGNLLRHIKLH<br>TGEKPFKCHLCNYACQRRDAL*

Supplementary Table 3: RNA and DNA sequences for genome editing

Type	Experiment description	Sequence
crRNA – TCR beta locus	Targeting the TCR beta locus to integrate the initial DROP-CAR	ACCGAAGUACUGUUCAUAAU
crRNA – LD3 locus -1	Targeting the LD3 site to introduce a frameshift mutation	GCCCUACAACUGCGUCUGAU
crRNA – LD3 clone F6-1	Targeting the LD3 site to repair the frameshift mutation	CACCGAUCAGACGCAGUUGU
crRNA – LD3 clone F6-2	Targeting the LD3 site to repair the frameshift mutation	ACCGAUCAGACGCAGUUUGU
crRNA – LD3 clone C8-1	Targeting the LD3 site to repair the frameshift mutation	CCCUACAAACUGCGUCUGAU
crRNA – LD3 clone C8-2	Targeting the LD3 site to repair the frameshift mutation	UCUGAUUGCCCUACACUGAU
ssODN – NNK1	Site saturation mutagenesis of A130 and R134	CATGCTGGGCCAATCGACGGAAGAACTGCGTGTCCGCCTGGCGAGCCATCTGATT**NNK**CTACAACTG**NNK**CTGATCGGTGACGCATTCGACCTACAGAAACGCCTGGCTGTCTACCAAGCAGGCG
ssODN – NNK2	Site saturation mutagenesis of Q132	CATGCTGGGCCAATCGACGGAAGAACTGCGTGTCCGCCTGGCGAGCCATCTGATTGCCCTA**NNK**CTGCGTCTGATCGGTGACGCATTCGACCTACAGAAACGCCTGGCTGTCTACCAAGCAGGCG
ssODN – NNK3	Site saturation mutagenesis of D138	GGAAGAACTGCGTGTCCGCCTGGCGAGCCATCTGATTGCCCTACAACTGCGTCTGATCGGT**NNK**GCATTCGACCTACAGAAACGCCTGGCTGTCTACCAAGCAGGCGCTGCTGAGAGCGCTAATT
ssODN – NNK4	Site saturation mutagenesis of A139	AGAACTGCGTGTCCGCCTGGCGAGCCATCTGATTGCCCTACAACTGCGTCTGATCGGTGAC**NNK**TTCGACCTACAGAAACGCCTGGCTGTCTACCAAGCAGGCGCTGCTGAGAGCGCTAATTGGA
LD3_oligo_F	PCR primer for genomic LD3 amplification (forward)	AAAGAACTACAGGCCGCACA
LD3_oligo_R	PCR primer for genomic LD3 amplification (reverse)	CGAACTGAGGGTGTGACCAG

Supplementary Table 4: Deep sequencing results after library sorts

Enriched variants are marked in red, undetected variants are marked in grey. Wild type residues are excluded from analysis. For the double mutant library NNK1, left to right refers to mutations of A130 and top to bottom to mutations of R134.

V5/Strep double positive (assembled CARs; sort 1)																					
		A	R	N	D	C	Q	E	G	H	I	L	K	M	F	P	S	T	W	Y	V
NNK1 A130X R134X	A	0.1352	0.0165	0	0	0	0	0.0002	0.4474	0	0	0	0	0	0.0019	0.0004	0.0068	0	0.0023	0.0015	0.0006
	R	X	0.0076	0	0.0038	0.0118	0.0004	0.0343	1.3922	0	0.0059	0.0009	0	0.0002	0.0006	0.1502	0.3079	0.0133	0.0205	0.0064	0.0631
	N	0	0	0	0	0	0	0	0.0004	0	0	0	0	0	0	0	0.0002	0	0	0	0
	D	0	0	0	0	0.0055	0	0	0	0	0	0	0	0	0	0	0.0019	0	0	0	0
	C	0.1678	0.0017	0.0040	0	0.0002	0	0.0004	0.3646	0	0	0	0	0	0	0.0055	0.0009	0	0.0002	0.0040	0.0038
	Q	0.0148	0	0	0.0002	0	0	0	0.0349	0	0	0	0	0	0	0.0099	0.0002	0	0	0	0
	E	0.0002	0	0	0	0.0004	0	0	0.0006	0	0	0	0	0	0	0	0	0	0	0	0
	G	0.2491	0.0055	0	0.0083	0.0076	0	0.0002	0.7957	0.0002	0.0085	0.0027	0	0.0021	0	0.0178	0.0011	0.0002	0	0	0.0008
	H	0.0142	0.0002	0	0	0	0.0006	0	0.0851	0	0	0.0082	0	0	0	0.0002	0.0053	0	0.0104	0	0
	I	0.0463	0	0	0.0004	0.0104	0	0	0.0008	0	0	0	0	0	0	0	0	0	0	0.0013	0.0002
	L	0.0169	0	0	0.0013	0.0019	0.0002	0.0068	0.0131	0	0.0021	0	0	0	0.0085	0	0.0193	0	0	0.0002	0.0080
	K	0.1253	0	0	0	0.0011	0	0.0002	0.2495	0	0	0.0038	0	0	0.0002	0.0436	0.0728	0	0	0	0.0222
	M	0.0468	0	0.0030	0	0	0.0074	0.0002	0.0844	0	0	0.0013	0	0	0	0.0015	0.0017	0	0	0	0
	F	0.0102	0.0072	0	0	0	0	0	0	0	0.0108	0.0021	0	0	0	0	0	0	0	0	0.0104
	P	0.0009	0	0	0	0	0	0	0.0025	0	0	0	0	0.0017	0	0	0.0002	0	0	0	0
	S	0.0607	0.0017	0	0.0032	0	0	0.0008	1.0589	0	0.0044	0.0061	0	0.0038	0	0.0013	0.0004	0.0015	0.0180	0	0.0157
	T	0.0087	0.0195	0	0.0002	0	0	0.0066	0.1115	0	0	0	0	0.0017	0.0028	0.0004	0.0076	0	0	0	0
	W	0.0008	0.0159	0	0	0	0	0	0.0002	0	0	0	0	0.0082	0	0	0	0	0.0051	0	0
	Y	0.0002	0.0245	0	0	0	0	0	0	0	0	0	0	0	0	0	0	0	0	0	0
	V	0.0436	0	0	0	0	0.0114	0.0066	0.1086	0.0051	0	0	0	0	0	0.0006	0.0137	0	0	0.0004	0.0002
NNK2 Q132X		3.5404	7.1066	1.4477	1.8442	1.8394	X	2.4766	4.8437	1.8318	1.3615	5.4871	2.2791	2.2658	2.0967	2.1395	5.1317	2.7119	1.7581	1.4409	4.1860
NNK3 D138X		0.0525	0.0315	0.0315	X	0.0415	0.0193	0.2527	0.1232	0.0078	0.0034	0.0214	0	0.0133	0.0053	0	0.0610	0.0087	0.0140	0.0072	0.0387
NNK4 A139X		X	0.6749	0.2389	0.4148	0.3832	0.2508	0.4448	1.2090	0.1594	0.3445	1.5209	0.1194	0.6992	0.5151	0.4323	1.4198	0.7811	0.7802	0.3949	1.0883

Second selection of GFP positive cells (functional CARs; sort 3)

		A	R	N	D	C	Q	E	G	H	I	L	K	M	F	P	S	T	W	Y	V
**NNK1 A130X R134X**	A	0.0017	0.0007	0	0	0	0	0	0.0705	0	0	0	0	0	0	0	0	0	0	0	0
	R	X	0	0	0.0056	0	0	0.0013	0.8391	0	0	0	0	0	0	0.0020	0.0053	0.0083	0.0003	0	0.0218
	N	0	0	0	0	0	0	0	0	0	0	0	0	0	0	0	0	0	0	0	0
	D	0	0	0	0	0	0	0	0	0	0	0	0	0	0	0	0	0	0	0	0
	C	0.0242	0	0.0003	0	0	0	0	0.0119	0	0	0	0	0	0	0.0003	0	0	0	0	0
	Q	0.0003	0	0	0	0	0	0	0.0010	0	0	0	0	0	0	0	0	0	0	0	0
	E	0	0	0	0	0	0	0	0.0003	0	0	0	0	0	0	0	0	0	0	0	0
	G	0.0139	0	0	0	0.0003	0	0	0.0486	0	0	0	0	0	0	0	0	0	0	0	0
	H	0.0103	0	0	0	0	0.0007	0	0.0010	0	0	0.0003	0	0	0	0	0	0	0	0	0
	I	0	0	0	0	0	0	0	0	0	0	0	0	0	0	0	0	0	0	0	0
	L	0.0023	0	0	0	0	0	0	0.0007	0	0	0	0	0	0	0	0.0003	0	0	0	0
	K	0.0023	0	0	0	0	0	0	0.0298	0	0	0	0	0	0	0.0007	0.0040	0	0	0	0
	M	0.0003	0	0	0	0	0	0	0.0010	0	0	0	0	0	0	0	0	0	0	0	0
	F	0	0.0003	0	0	0	0	0	0	0	0	0	0	0	0	0	0	0	0	0	0
	P	0.0010	0	0	0	0	0	0	0.0063	0	0	0	0	0	0	0	0	0	0	0	0
	S	0.0705	0	0	0.0003	0	0	0.0013	2.2738	0	0	0	0	0	0	0	0	0	0	0	0.0010
	T	0.0007	0	0	0	0	0	0	0.0026	0	0	0	0	0	0	0	0	0	0	0	0
	W	0	0	0	0	0	0	0	0	0	0	0	0	0.0003	0	0	0	0	0	0	0
	Y	0	0	0	0	0	0	0	0	0	0	0	0	0	0	0	0	0	0	0	0
	V	0.0013	0	0	0	0	0	0	0.0308	0	0	0	0	0	0	0	0	0	0	0	0
**NNK2 Q132X**		1.3407	4.3928	1.9270	1.1707	0.5939	X	2.3079	3.9997	0.5873	0.7230	5.8417	1.2114	4.0076	5.1981	2.7794	4.4712	2.5918	3.6049	0.9635	2.2265
**NNK3 D138X**		0.0020	0.0003	0.0116	X	0	0	0.0083	0.0685	0.0003	0	0.0007	0	0	0	0	0.0007	0	0.0003	0.0017	0.0056
**NNK4 A139X**		X	0.2601	1.8298	1.2653	1.5816	0.0725	0.1158	3.8217	0.0030	0.4583	4.1837	0.0026	0.0513	0.2842	0.0490	0.4586	1.0267	0.3349	1.3616	0.7001

Second drug-sensitivity selection; sort 5

		A	R	N	D	C	Q	E	G	H	I	L	K	M	F	P	S	T	W	Y	V
**NNK1 A130X R134X**	A	0.0472	0	0	0	0	0	0	0.0079	0	0	0	0	0	0	0	0	0	0	0	0
	R	X	0	0	0.0018	0	0	0.0240	0.0797	0	0	0	0	0	0	0.0011	0.0053	0.0061	0	0	0.0087
	N	0	0	0	0	0	0	0	0.0011	0	0	0	0	0	0	0	0	0	0	0	0
	D	0	0	0	0	0	0	0	0	0	0	0	0	0	0	0	0	0	0	0	0
	C	0.0061	0.0008	0.0003	0	0	0	0	0.1275	0	0	0	0	0	0	0	0	0	0	0	0
	Q	0	0	0	0	0	0	0	0	0	0	0	0	0	0	0	0	0	0	0	0
	E	0	0	0	0	0	0	0	0	0	0	0	0	0	0	0	0.0005	0	0	0	0
	G	0.0058	0	0	0.0011	0	0	0	0.2827	0	0	0	0	0	0	0	0	0	0	0	0

	H	0.0024	0	0	0	0	0	0	0	0	0	0	0	0	0	0	0	0	0.0003	0	0
	I	0.0005	0	0	0	0	0	0	0	0	0	0	0	0	0	0	0	0	0	0	0
	L	0.0013	0	0	0	0	0	0	0.0011	0	0	0	0	0	0.0003	0	0.0003	0	0	0	0
	K	0.0018	0	0	0	0	0	0	0.0011	0	0	0	0	0	0	0.0003	0.0457	0	0	0	0
	M	0.0003	0	0	0	0	0	0	0.0013	0	0	0	0	0	0	0	0	0	0	0	0
	F	0	0	0	0	0	0	0	0	0	0	0	0	0	0	0	0	0	0	0	0
	P	0.0003	0	0	0	0	0	0	0.0140	0	0	0	0	0	0	0	0	0	0	0	0
	S	0.0628	0.0037	0	0	0	0	0.0011	5.3266	0	0	0	0	0	0	0	0.0003	0	0.0003	0	0.0005
	T	0.0003	0	0	0	0	0	0	0.0058	0	0	0	0	0	0	0	0	0	0	0	0
	W	0	0	0	0	0	0	0	0	0	0	0	0	0	0	0	0	0	0	0	0
	Y	0	0.0003	0	0	0	0	0	0.0008	0	0	0	0	0	0	0	0	0	0	0	0
	V	0.0185	0	0	0.0003	0	0.0005	0	1.0879	0	0	0	0	0	0	0	0.0003	0	0	0	0
**NNK2 Q132X**		2.5742	0.2227	0.3162	1.1216	2.7975	X	2.0944	7.4670	0.0852	1.8300	12.0227	0.0433	8.4300	5.4839	11.8844	8.1374	1.9150	3.5006	0.4231	4.4008
**NNK3 D138X**		0.0003	0	0.0026	X	0.0003	0.0003	0.0032	0.0248	0.0003	0	0.0005	0	0.0003	0	0	0	0.0003	0	0	0.0013
**NNK4 A139X**		X	2.2525	0.0185	0.0367	0.1180	0.0029	0.0238	3.6386	0.0018	0.0137	0.0953	0.0008	0.0090	0.2280	0.0892	0.0179	0.0322	0.0206	1.2650	0.0705

Third drug-sensitivity selection; sort 6 - population V5-medium

		A	R	N	D	C	Q	E	G	H	I	L	K	M	F	P	S	T	W	Y	V
**NNK1 A130X R134X**	A	0.0541	0	0	0	0	0	0.0004	0.0021	0	0	0	0	0	0	0	0	0	0	0	0
	R	X	0	0	0.0017	0	0	0.0107	0.0689	0	0	0	0	0	0	0	0.0021	0.0017	0	0	0.0017
	N	0	0	0	0	0	0	0	0.0004	0	0	0	0	0	0	0	0	0	0	0	0
	D	0	0	0	0	0	0	0	0	0	0	0	0	0	0	0	0	0	0	0	0
	C	0.0012	0	0	0	0	0	0	0	0	0	0	0	0	0	0	0	0	0	0	0
	Q	0	0	0	0	0	0	0	0	0	0	0	0	0	0	0	0	0	0	0	0
	E	0	0	0	0	0	0	0	0.0050	0	0	0	0	0	0	0	0	0	0	0	0
	G	0.0726	0	0	0.0033	0.0004	0	0	6.1655	0	0	0	0	0	0	0	0.0054	0	0	0	0.0012
	H	0.0021	0	0	0	0	0	0	0	0	0	0	0	0	0	0	0	0	0	0	0
	I	0	0	0	0	0	0	0	0	0	0	0	0	0	0	0	0	0	0	0	0
	L	0.0012	0	0	0	0	0	0	0.0008	0	0	0	0	0	0	0	0	0	0	0	0
	K	0	0	0	0	0	0	0	0.0017	0	0	0	0	0	0	0	0.0553	0	0	0	0
	M	0	0	0	0	0	0	0	0.0008	0	0	0	0	0	0	0	0	0	0	0	0
	F	0	0	0	0	0	0	0	0	0	0	0	0	0	0	0	0	0	0	0	0
	P	0.0008	0	0	0	0	0	0	0.0128	0	0	0	0	0	0	0	0	0	0	0	0
	S	0.0933	0	0	0.0004	0	0	0.0004	6.2679	0	0	0	0	0	0	0	0.0045	0	0	0	0.0025
	T	0.0004	0	0	0	0	0	0	0.0041	0	0	0	0	0	0	0	0	0	0	0	0

	W	0.0004	0	0	0	0	0	0	0.0008	0	0	0	0	0	0	0	0	0	0	0	0
	Y	0	0	0	0	0	0	0	0.0012	0	0	0	0	0	0	0	0	0	0	0	0
	V	0.0062	0	0	0	0	0	0.0004	0.7623	0	0	0	0	0	0	0	0.0008	0	0	0	0
**NNK2 Q132X**		1.7513	0.1094	0.1461	0.0466	7.3546	X	0.0037	0.4222	0.0301	0.3521	18.8998	0.0029	0.9485	3.2177	10.9591	0.1255	0.0900	0.4573	0.0277	8.0575
**NNK3 D138X**		0.0008	0	0.0017	X	0	0	0.0021	0.0140	0.0004	0	0	0	0	0	0	0	0	0	0	0.0012
**NNK4 A139X**		X	19.5870	0.0012	0.0008	0.0017	0.0017	0.0041	8.5252	0.0012	0	0.0008	0.0025	0.0037	0.0598	0.2055	0.0054	0.0025	0.0037	0.3735	0.0062

Third drug-sensitivity selection; sort 6 - population V5-low

		A	R	N	D	C	Q	E	G	H	I	L	K	M	F	P	S	T	W	Y	V
**NNK1 A130X R134X**	A	0.0095	0	0	0	0	0	0.0003	0.1447	0	0	0	0	0	0	0	0.0008	0	0	0	0
	R	X	0	0	0.0008	0	0	1.4730	0.0767	0	0	0	0	0	0	0	0.0003	0.0003	0	0	0
	N	0	0	0	0	0	0	0	0	0	0	0	0	0	0	0	0	0	0	0	0
	D	0	0	0	0	0	0	0	0	0	0	0	0	0	0	0	0	0	0	0	0
	C	0.0005	0	0	0	0	0	0.0015	0.0003	0	0	0	0	0	0	0	0	0	0	0	0
	Q	0	0	0	0	0	0	0	0	0	0	0	0	0	0	0	0	0	0	0	0
	E	0	0	0	0	0	0	0	0.0061	0	0	0	0	0	0	0	0	0	0	0	0
	G	0.0025	0	0	0.0005	0	0	0.0005	0.3610	0	0	0	0	0	0	0	0.0018	0	0	0	0.0005
	H	0.0003	0	0	0	0	0	0.0010	0	0	0	0	0	0	0	0	0	0	0	0	0
	I	0	0	0	0	0	0	0	0	0	0	0	0	0	0	0	0	0	0	0	0
	L	0.0030	0	0	0	0	0	0.0013	0.0127	0	0	0	0	0	0	0	0	0	0	0	0
	K	0	0	0	0	0	0	0	0	0	0	0	0	0	0	0	0	0	0	0	0
	M	0.0003	0	0	0.0003	0	0	0	0.0386	0	0	0	0	0	0	0	0	0	0	0	0
	F	0	0	0	0	0	0	0	0	0	0	0	0	0	0	0	0	0	0	0	0
	P	0	0	0	0	0	0	0.0008	0.0008	0	0	0	0	0	0	0	0	0	0	0	0
	S	0.0020	0	0	0	0	0	0.0003	0.1764	0	0	0	0	0	0	0	0	0	0	0	0.0003
	T	0	0	0	0	0	0	0	0	0	0	0	0	0	0	0	0	0	0	0	0
	W	0	0	0	0	0	0	0	0	0	0	0	0	0	0	0	0	0	0	0	0
	Y	0	0	0	0	0	0	0	0	0	0	0	0	0	0	0	0	0	0	0	0
	V	0.4923	0.0010	0	0.0312	0.0038	0	0.0427	71.8158	0	0	0	0	0	0	0	0.0350	0.0005	0	0	0.0033
**NNK2 Q132X**		0.0307	0.0030	0.0003	0	0.1170	X	0.0003	0.0206	0.0003	0.0536	3.3018	0	0.0498	0.0724	0.1203	0.0310	0.0005	0.0053	0.0008	0.1858
**NNK3 D138X**		0	0	0.0008	X	0	0	0	0.0028	0	0	0	0	0	0	0	0	0	0	0	0
**NNK4 A139X**		X	19.9162	0.0008	0	0.0005	0.0005	0	0.2381	0	0	0.0003	0.0074	0.0015	0.0008	0.0013	0.0074	0.0048	0.0015	0.0416	0.0003

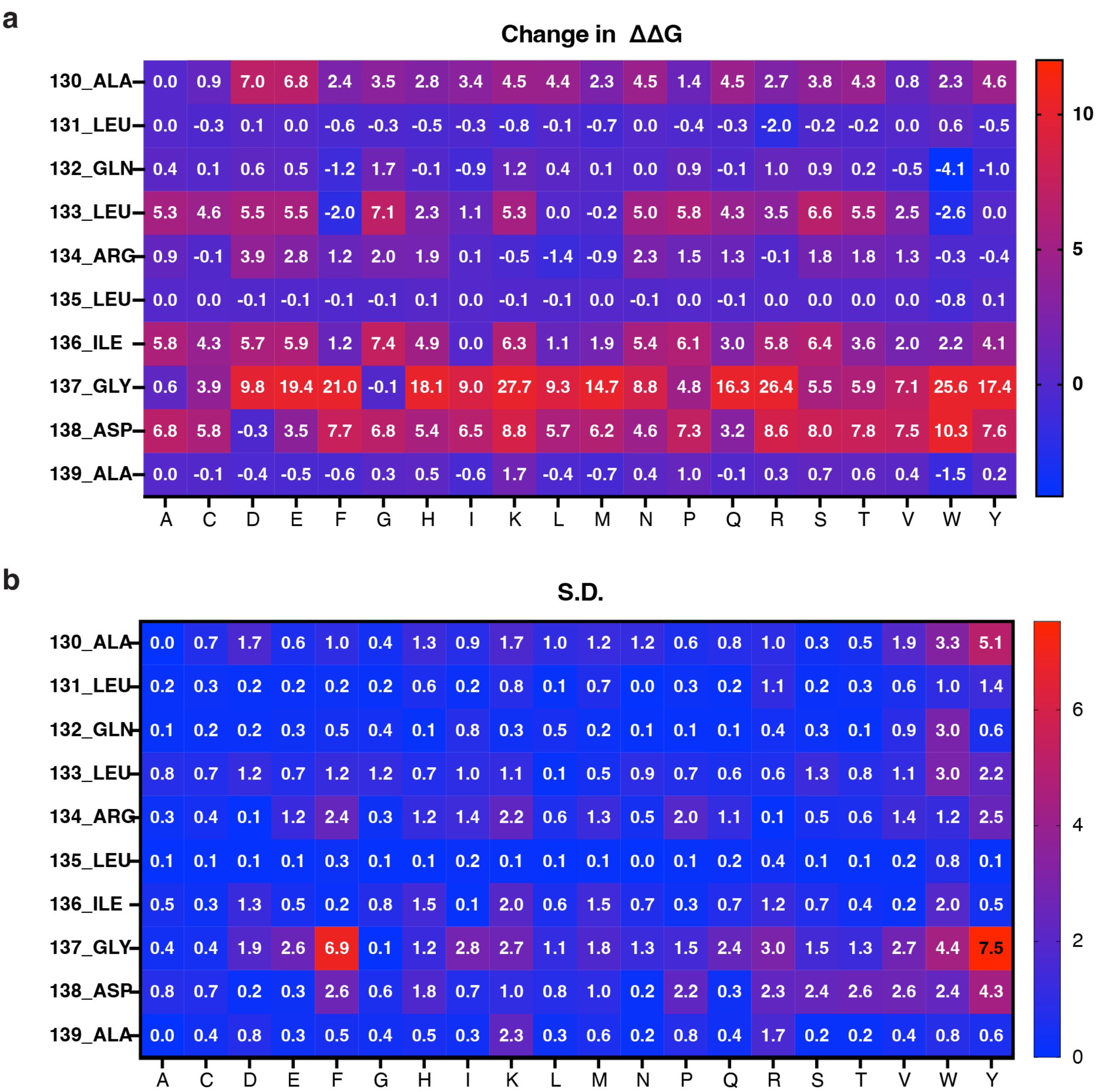

Supplementary Figure 1: In Silico Site Saturation Mutagenesis.

a, b, Heat map for the mean values and standard deviation of changes in Gibbs free energy (ΔΔG) in Rosetta energy units resulting from site saturation mutagenesis of interface residues predicted by Rosetta rigid-body docking. The residues chosen for experimental mutagenesis are shown in bold on the Y-axis, and possible amino acid replacements on the X-axis. S.D. = standard deviation.

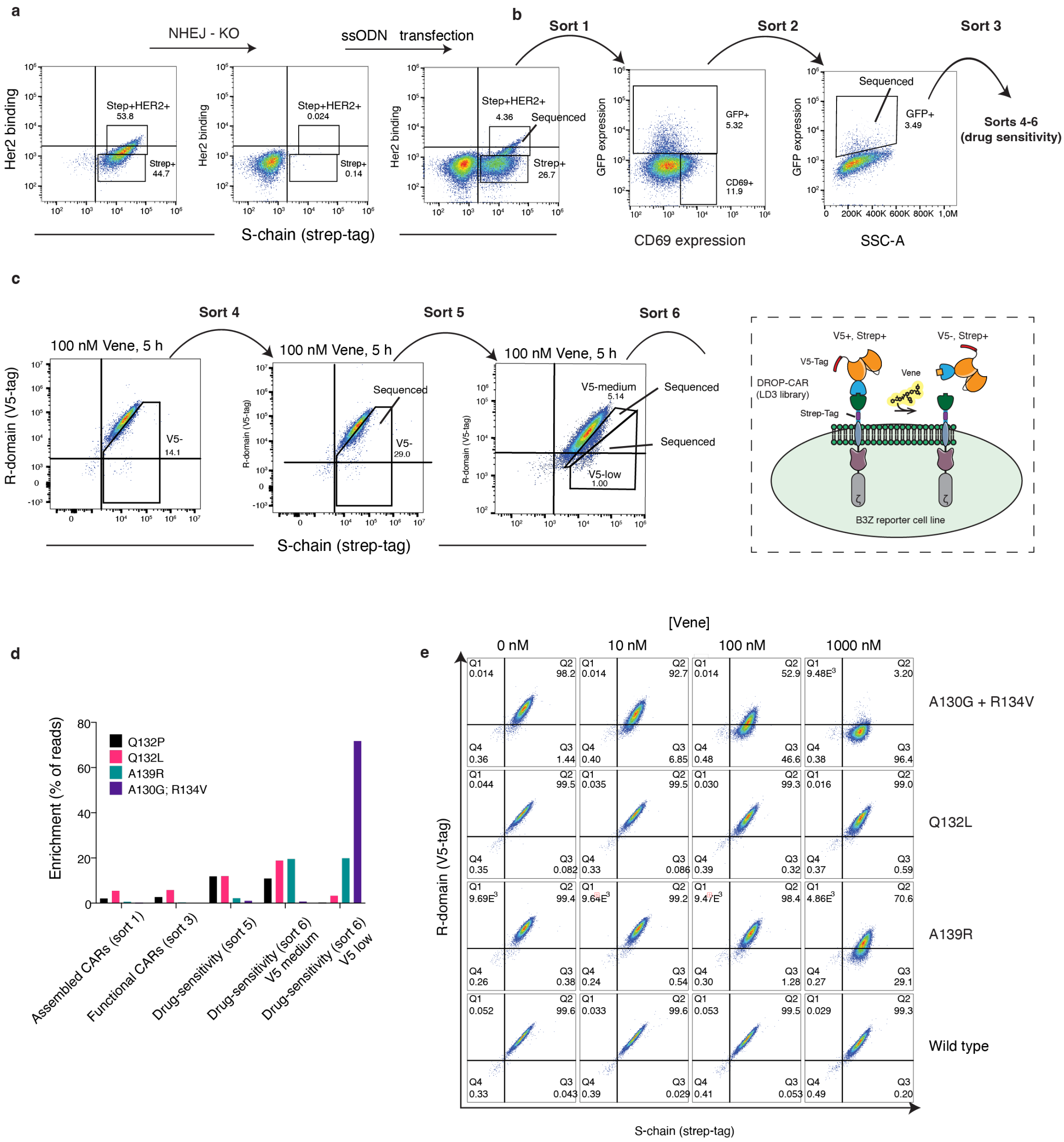

f

```
Range 1: 23 to 166
Identities:132/144(92%), Positives:135/144(93%), Gaps:0/144(0%)

Query  1    GQRWELALGRFLEYLSWVSTLSEQVQEELLSSQVTQELRALMDETMKELKAYKSELEEQL  60
            GQRWELALGRF +YL WV TLSEQVQEELLSSQVTQELRALMDETMKELKAYKSELEEQL
Sbjct  23   GQRWELALGRFWDYLRWVQTLSEQVQEELLSSQVTQELRALMDETMKELKAYKSELEEQL  82

Query  61   TPVAEETRARLSKELQAAQARLGADMEDVRGRLVQYRGEVQAMLGQSTEELRVRLASHLI  120
            TPVAEETRARLSKELQAAQARLGADMEDVRGRLVQYRGEVQAMLGQSTEELRVRLASHL
Sbjct  83   TPVAEETRARLSKELQAAQARLGADMEDVRGRLVQYRGEVQAMLGQSTEELRVRLASHLR  142

Query  121  GLQLVLIGDAFDLQKRLAVYQAGA  144
             L+   L+ DA DLQKRLAVYQAGA
Sbjct  143  KLRKRLLRDADDLQKRLAVYQAGA  166
```

Supplementary Figure 2: Library generation and DROP-CAR functionality sorts.
a, From the initial Cas9 expressing B3Z-Drop-CAR cell line, CAR expressing cells were treated with a gRNA that cuts in the LD3 region and is repaired by nonhomologous end joining (NHEJ), causing frameshift mutations. The sorted cell population without CAR expression was transfected with the ssODN library to integrate the variant library into the genomic LD3 site by homology-directed repair (HDR). **b,**The population with high expression of fully assembled DROP-CARs (costained for the V5 tag on the R-domain and the Strep tag on the

transmembrane S-chain) was sorted and used for coculture experiments with $Her2^+$ SKOV3 cells. High xpression of the reporter gene GFP was used to enrich cells expressing functional DROP-CARs on their surface. The functional sort was conducted twice, followed by the drug sensitivity screening with venetoclax. **c,** Drug sensitive DROP-CARs were enriched in 3 consecutive screens (gating shown for sorted cells, sorts 4-6). Weaker V5 staining in the presence of venetoclax indicates DROP-CAR disassembly. Populations chosen for deep sequencing are indicated. **Right:** schematic of R-domain dissociation in the presence of Venetoclax. The last panel is also shown in Figure 1h in the main manuscript. **d,** Enrichment of clones comprising more than 10% of the entire population after sorts as indicated. Deep sequencing results are listed in Table S4. **e,** The top hits of the enriched sequences from the V5-low and V5-medium populations of the third drug-sensitivity sort were analyzed after venetoclax treatment (5 h incubation). A decrease in the population for V5-tag staining in response to venetoclax indicates successful venetoclax-induced CAR disassembly. (The first and last panels shown for the wild type LD3 are the same as in **a**). **f,** Alignment of the mutant LD3 A130G; R134V (Query) to human ApoE4 (PDB 1GS9, UniProt ID P02649) via NCBI Protein BLAST. All differences compared to the human protein are highlighted. The numbering of the mutants in the main text is based on the crystal structure PDB 6IWB, whereas the alignment starts with the first residue used in the DROP-CAR. Accordingly, the sites of the double mutant correspond to A121G; R125V in the alignment. Range 1 refers to the aligned region of ApoE4. (Venetoclax; Vene)

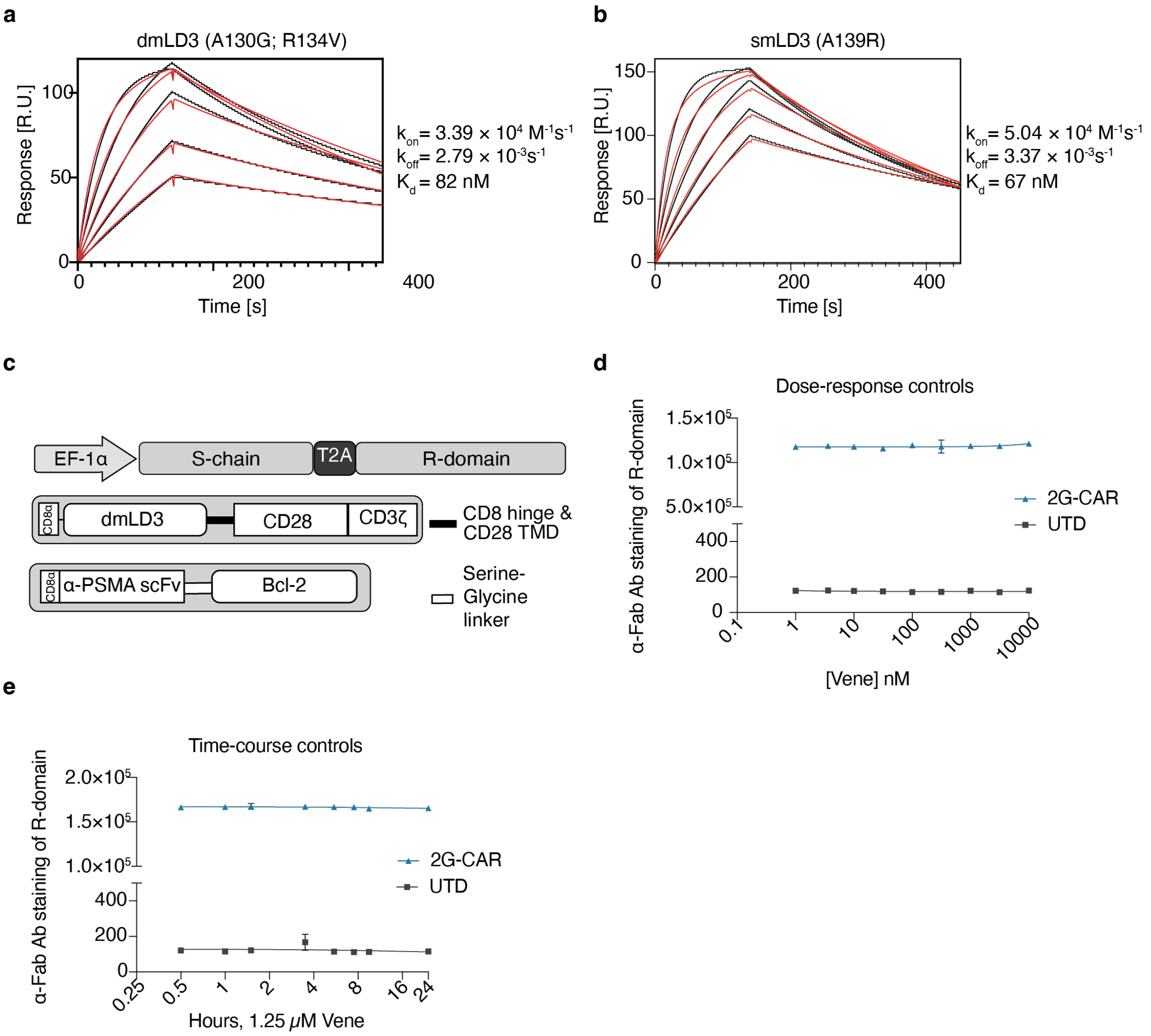

Supplementary Figure 3: Affinity characterization of LD3 variants enriched from the library screening for Bcl-2 and evaluation of optimized DROP-CAR expression in Jurkat cells.

a, Surface plasmon resonance (SPR) analysis of the dmLD3 variant (A130G+R134V) and Bcl-2. **b,** SPR analysis of the single (s)LD3 variant (A139R) and Bcl-2. **c,** Schematic of the α-PSMA DROP-CAR lentiviral expression cassette. **d,** Positive (second generation (2G)-CAR) and negative (untransduced; UTD) controls for the dose-response experiment of Jurkat DROP-CAR (comprising dmLD3; A130G+R134V) disruption **e,** Positive and negative controls for the time-course experiment of DROP-CAR Jurkat (comprising dmLD3) disruption.

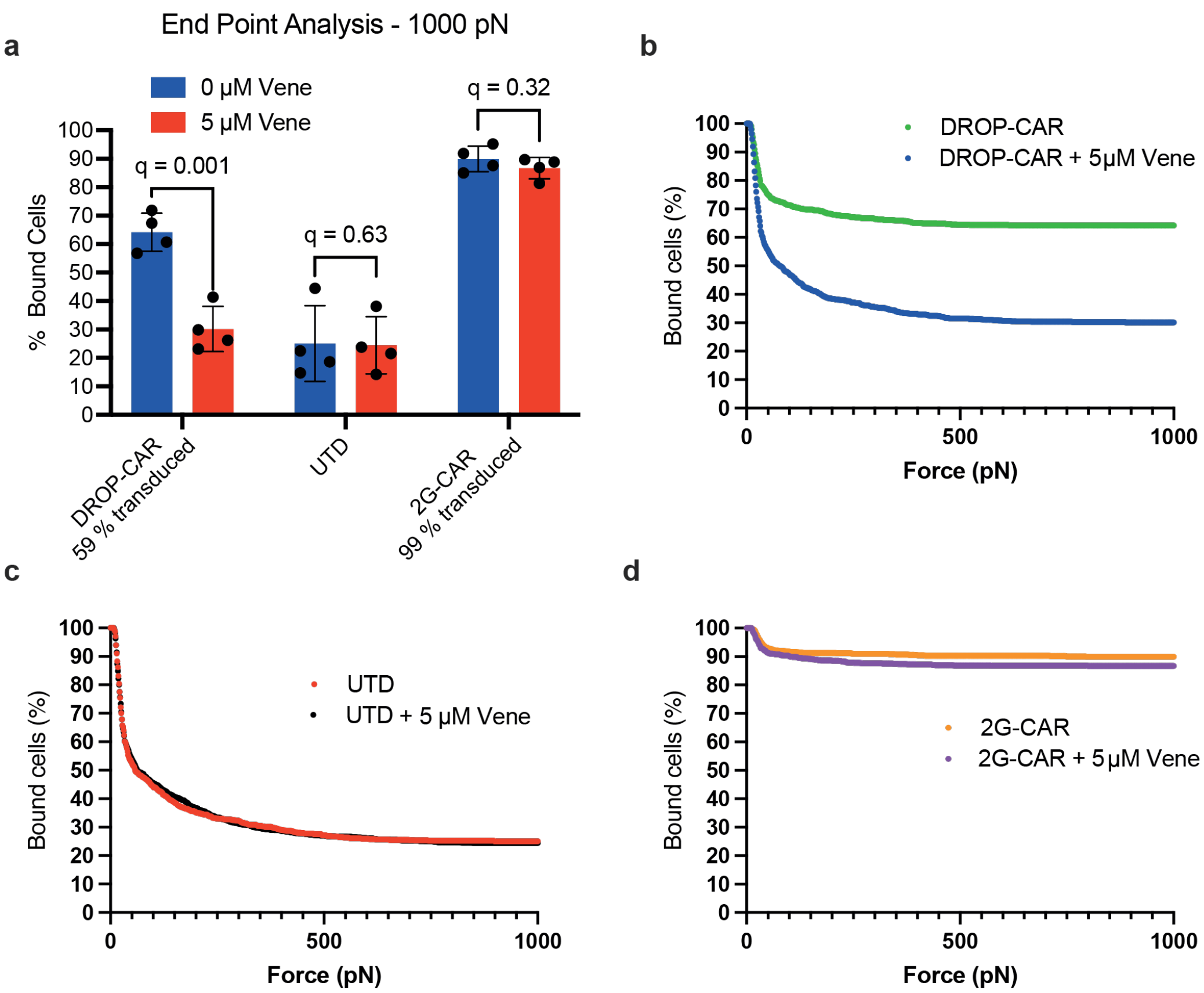

Supplementary Figure 4: Cell avidity analysis with hinge cells counted as bound.
a, Percentage of cells that remain bound after 2.5 min of a linear acoustic force ramp from 0 to 1000 pN. Partly detached cells (hinge cells) are counted as bound. Values are the mean ± s.d. of n = 4 measurements on separate chips. **b,c,d,** Percent of cells bound for the DROP-Jurkat cells, untransduced cells (UTD), and positive control second generation (2G) CAR-T cells over 2.5 min of a linear acoustic force ramp from 0 to 1000 pN. Transduction efficiency for the DROP CAR was 59% and for the 2G CAR 99%. Partly detached cells (hinge cells) are counted as bound. Values are n = 4 measurements on separate chips. (Venetoclax; Vene)

a

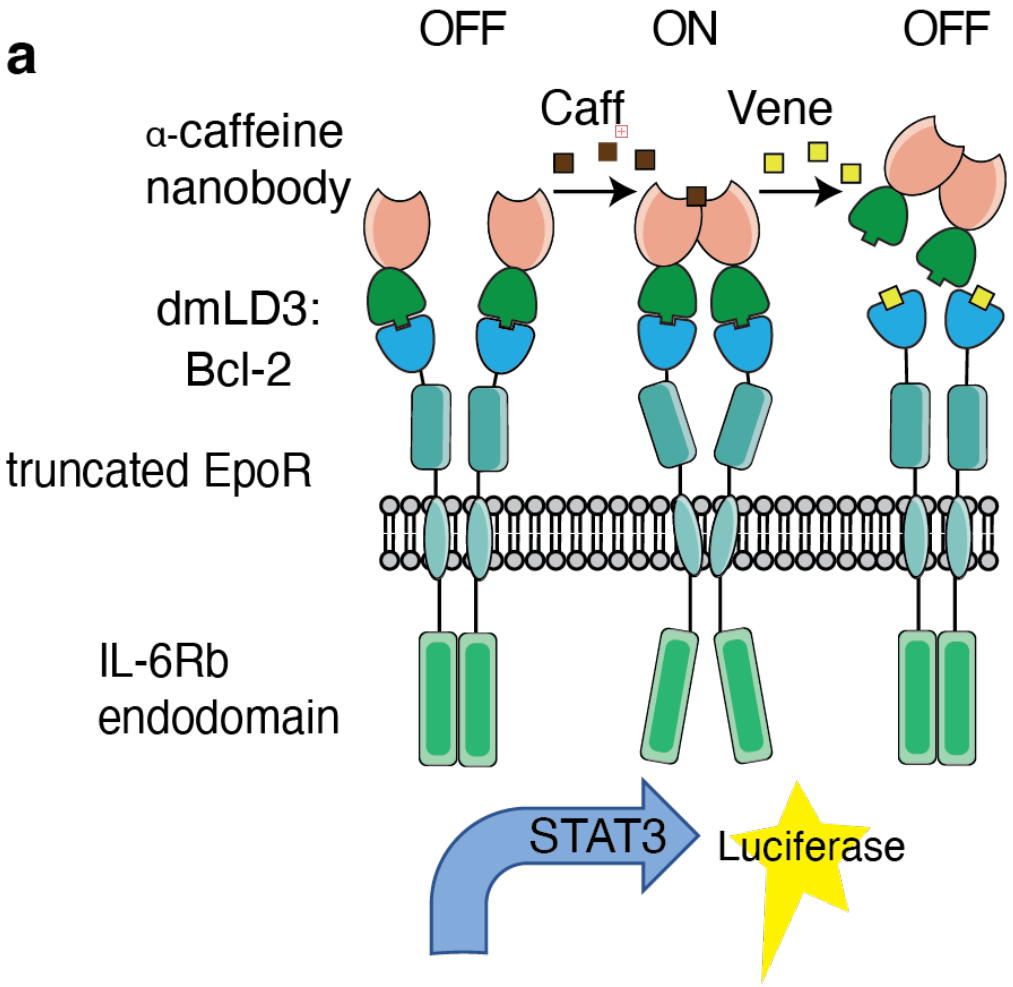

b

A	B	A AND (NOT B)
0	0	0
0	1	0
1	0	1
1	1	0

Caff	Vene	Caff AND (NOT Vene)
0	0	0
0	1	0
1	0	1
1	1	0

c

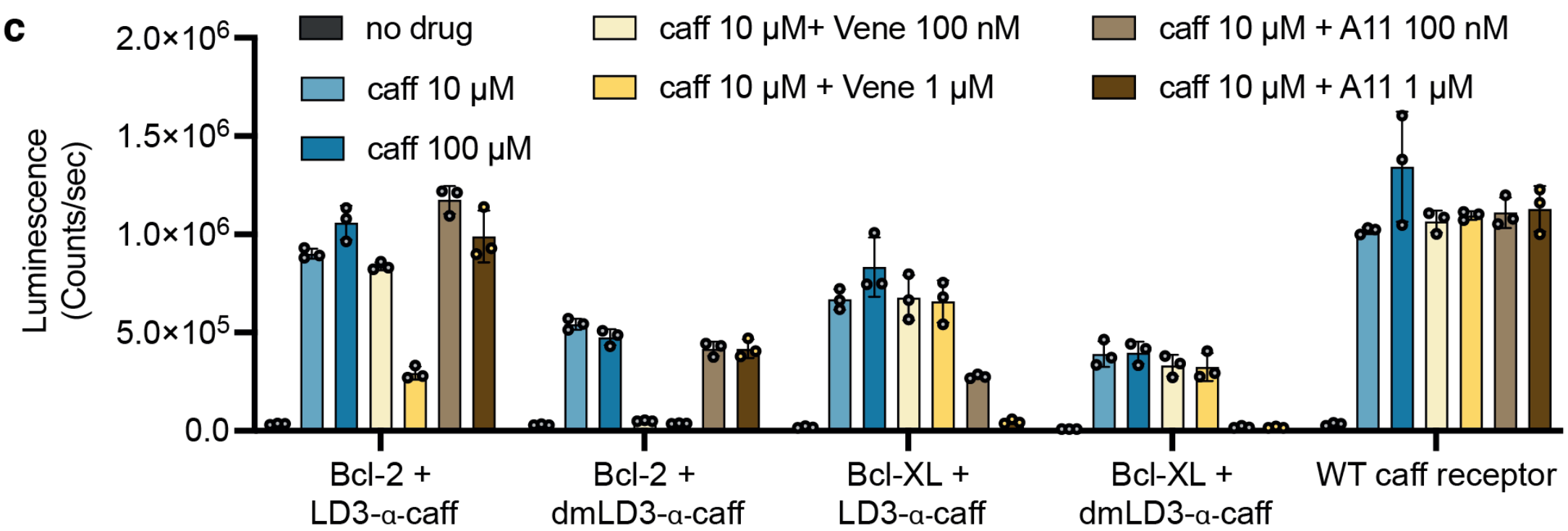

Supplementary Figure 5: Adapting the DROP design to the generalized extracellular molecule sensor (GEMS) cytokine receptor platform.

a, Schematic of engineered cytokine receptors that switch-on STAT3 signaling measured by STAT3-induced luciferase expression in response to caffeine, and switch-off in response to venetoclax. **b,** Truth table for A AND (NOT B), and for caffeine AND (NOT venetoclax). This is equivalent to A NIMPLY B. The same logic applies to the DROP-CAR for antigen AND (NOT venetoclax). **c,** Reporter gene expression in HEK293T cells to test Caffeine-ON, Venetoclax-OFF DROP-GEMS. Values are the mean ± s.d. of n = 3 biological replicates. (Caffeine; caff, Venetoclax; Vene)

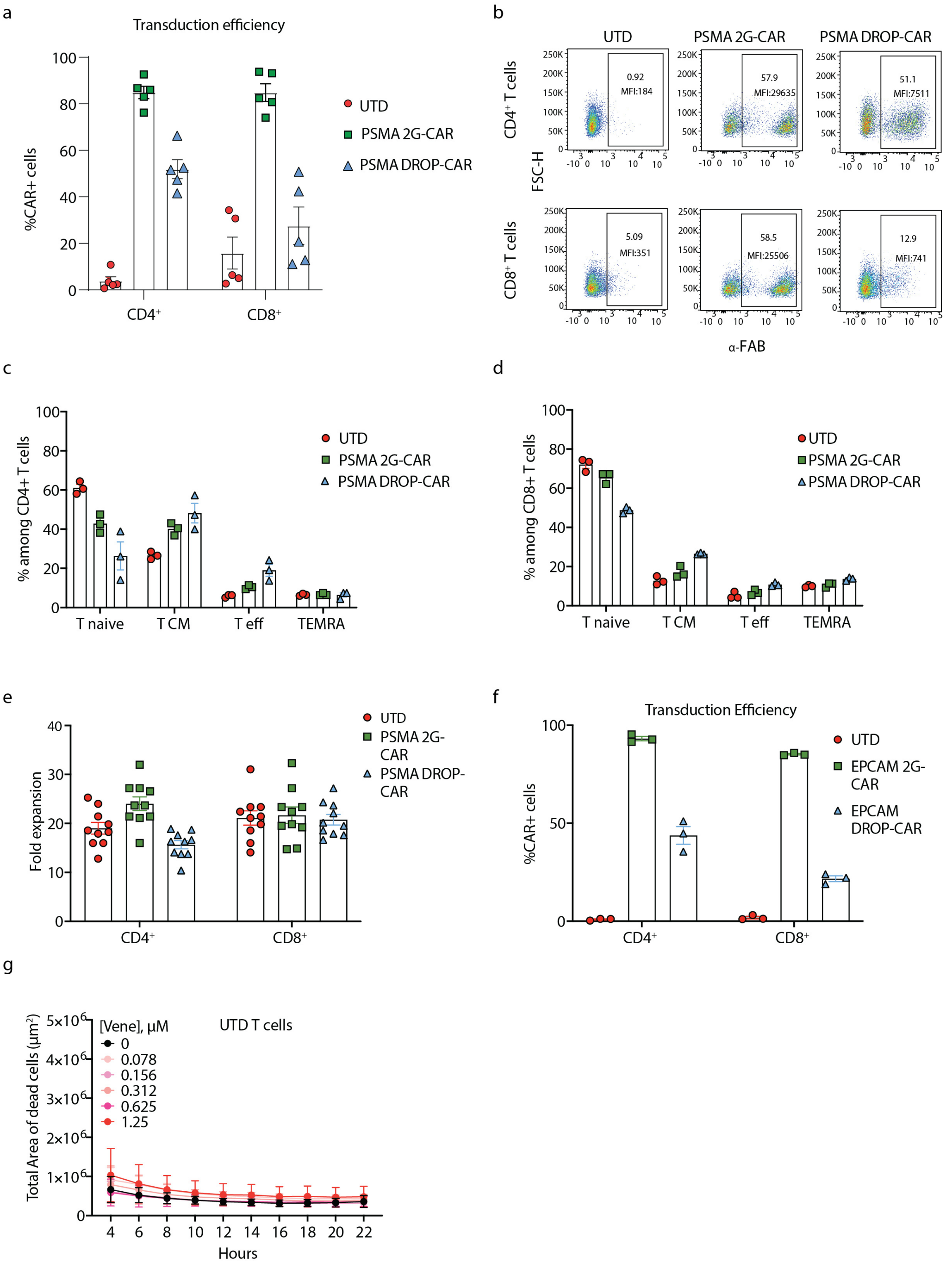

Supplementary Figure 6: DROP-CAR expression in primary human T cells.

a, b, Anti-PSMA second generation (2G) versus DROP-CAR expression levels in $CD8^+$ and $CD4^+$ human T cells compared to untransduced (UTD) cells to determine background staining. Measured by staining the scFv of the CAR and flow cytometric analysis. **c, d** Phenotype (T_{CM}; central memory T cells, T_{eff}; effector T cells, T_{EMRA}; terminally differentiated effector memory cells re-expressing CD45RA) for human $CD4^+$ (c) and $CD8^+$ (d) T cells expressing a 2G CAR or DROP-CAR versus UTD cells. **e**, Expansion of 2G- and DROP-CAR versus UTD primary human T-cells 11 days post-transduction. **f**, Anti-EpCAM 2G versus DROP-CAR expression levels in $CD8^+$ and $CD4^+$ human T cells. **g** UTD cells as negative control for the same human donor derived cells as in Figure 5 e, f. Values are the mean ± s.e.m. of n = 3 (c, d, f, g), n = 5 (a), n = 10 (e) human donors.

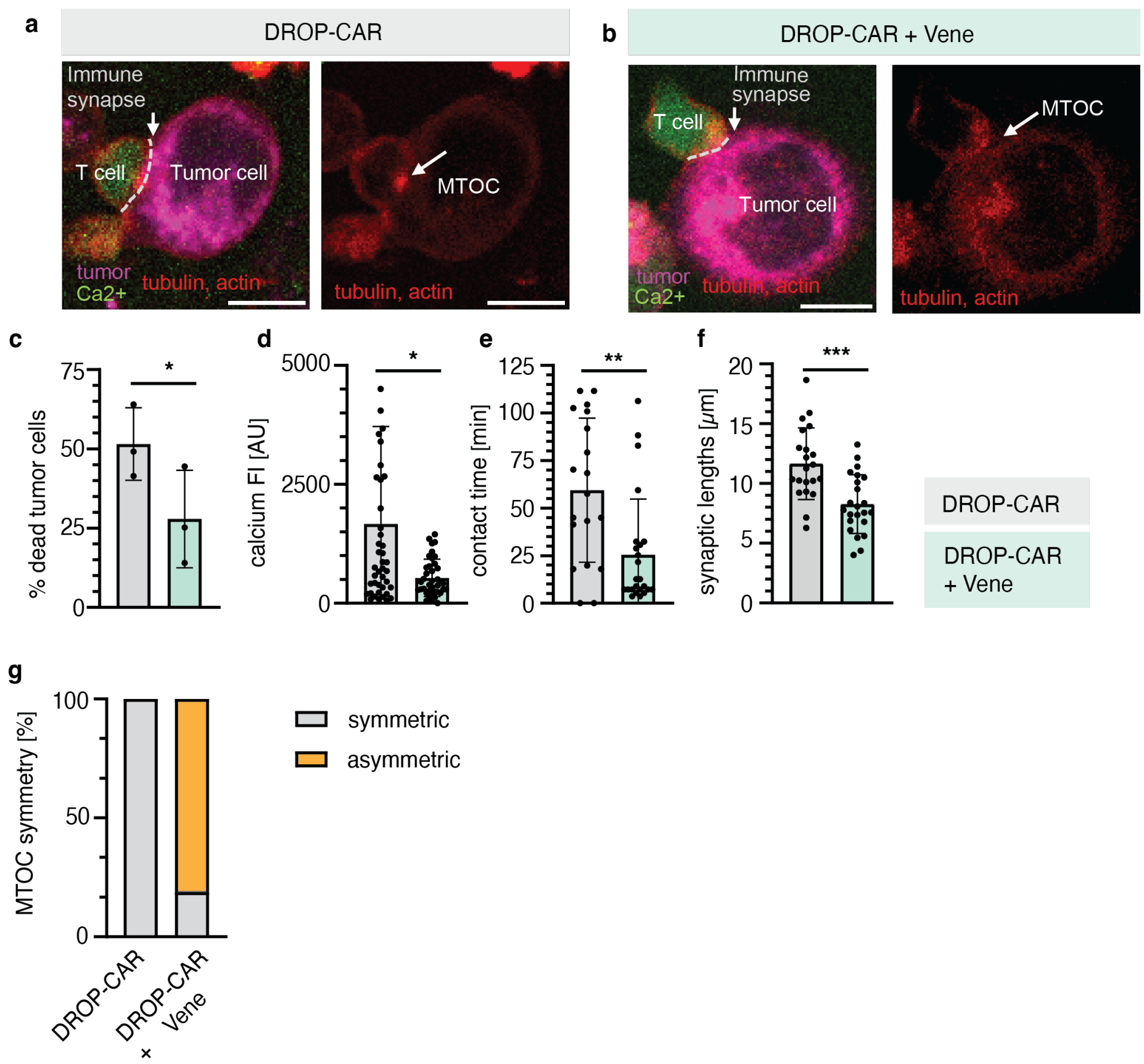

Supplemental Figure 7: Confocal live microscopy of DROP-CAR T cells and target tumor cells in the absence or presence of Venetoclax.

a,b, Representative stills from full visual fields at timepoint 0. T cells are labelled for calcium influx (green), actin and tubulin (red) to visualize the major microtubule organizing center (MTOC), and carboxyfluorescein succinimidyl ester (CFSE; magenta). The MTOC position indicates T cell cytoskeletal polarization upon immune synapse (IS) formation. **a,** DROP-CAR T cells (grey) form functional immune synapses with tumor cells, showing MTOC translocation toward the centre of the IS. **b,** Exposure to Venetoclax (light green) results in short-lived T cell–tumor cell contacts with asymmetric MTOC positioning. **c,** Quantification of tumor cell death after 6 hours of co-culture, expressed as a percentage of total tumor cells per visual field. Data are mean +/- S.D, n=3 HDs. Two-sided paired t-test*, **P= 0.0447. **d,** Fluorescence intensity of the calcium influx signal at the start of imaging, measured from maximum intensity projections of three z-stacks. Background-corrected integrated intensity was quantified at three locations per T cell. Each data point represents one cell. Data are mean +/- S.D, n=3 HDs. Two sides Mann Whitney, **P= 0.0148. **e,** T cell–tumor cell contact time at immune synapses, expressed in minutes. Each data point represents one cell. Data are mean +/- S.D, n=2 HDs. Two sides of the Mann-Whitney *P=0.0035. **f,** Length of the synaptic interface measured at the moment of MTOC polarization, expressed in μm. Each data point represents one cell. Data are mean +/- S.D, n=2 HDs.T-test two tailed, ***P= 0.0002 (normal distribution). **g,** MTOC positioning in cells with polarized MTOCs, classified as symmetric (within the central 33% of the synaptic interface) or asymmetric (within the outer 2 × 33.3%), n=2 HDs.

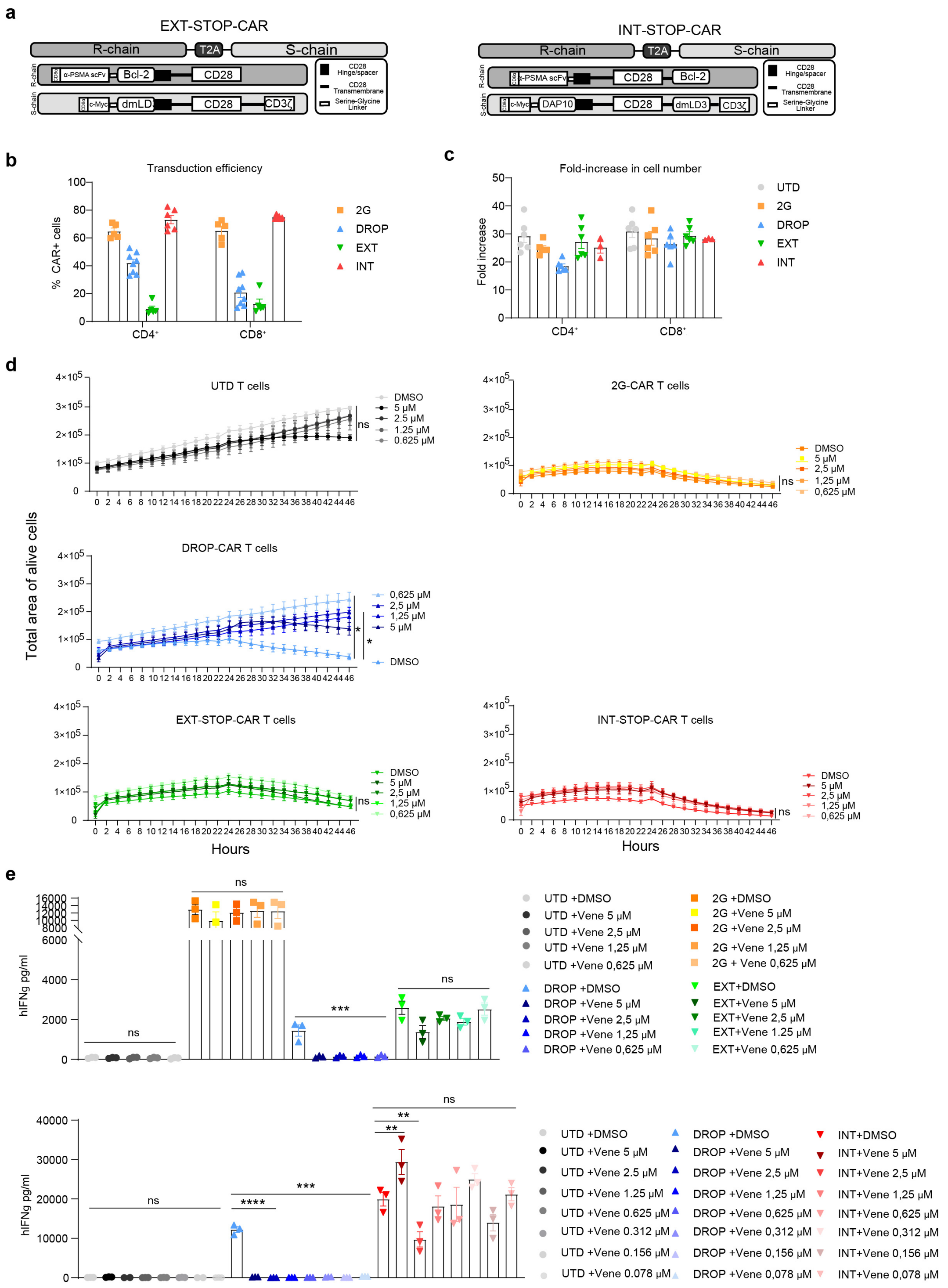

Supplemental Figure 8: In vitro characterization of STOP-CAR T cells comprising the dmLD3:Bcl-2 off-switch in the extracellular or intracellular region of the transmembrane R- and S-chains.

a, Left: schematic of STOP-CARs in which the optimized dmLD3:Bcl-2 off-switch has been incorporated into the extracellular (EXT) or intracellular (INT) region of the S- and R-chains. **b,** Fold growth rate of untransduced (UTD) as well as 2G-, DROP-, EXT-STOP- and INT-

STOP CAR T cells at day +12 post-transduction (n = 6; n = 4 HDs). **c,** Transduction efficiency of 2G-, DROP-, EXT-STOP- and INT-STOP CAR T cells at day +12 post-transduction (n = 6; n = 3 HDs). **d,** IncuCyte cytotoxicity assay at an effector to target ratio of 2:1 to evaluate PC3-PIP tumor cell killing by anti-PSMA 2G-, DROP-, EXT-STOP and INT-STOP CAR T cells in the presence of up to 5 μM venetoclax (n = 3 HDs). Two-way ANOVA test was used to determine statistical significance by comparing all groups. **e,** IFN-γ production by anti-PSMA CAR T cells upon 24 hours coculture with $PSMA^+$ PC3-PIP cells +/- venetoclax (ven) at different concentrations (n = 3 HDs). One-way ANOVA test was used to determine statistical significance by comparing each CAR group separately (EXT) or all groups simultaneously (INT). Before all functional assays, the engineered T cells were rested and adjusted by mixing them with UTD cells for equivalent transgene expression levels. A mix of 50% $CD4^+$ T cells and 50% $CD8^+$ T cells was used in all functional experiments. (Vene; Venetoclax).). *$P < 0.05$, **$P < 0.01$, ***$P < 0.001$ and ****$P < 0.0001$. ns, not significant.

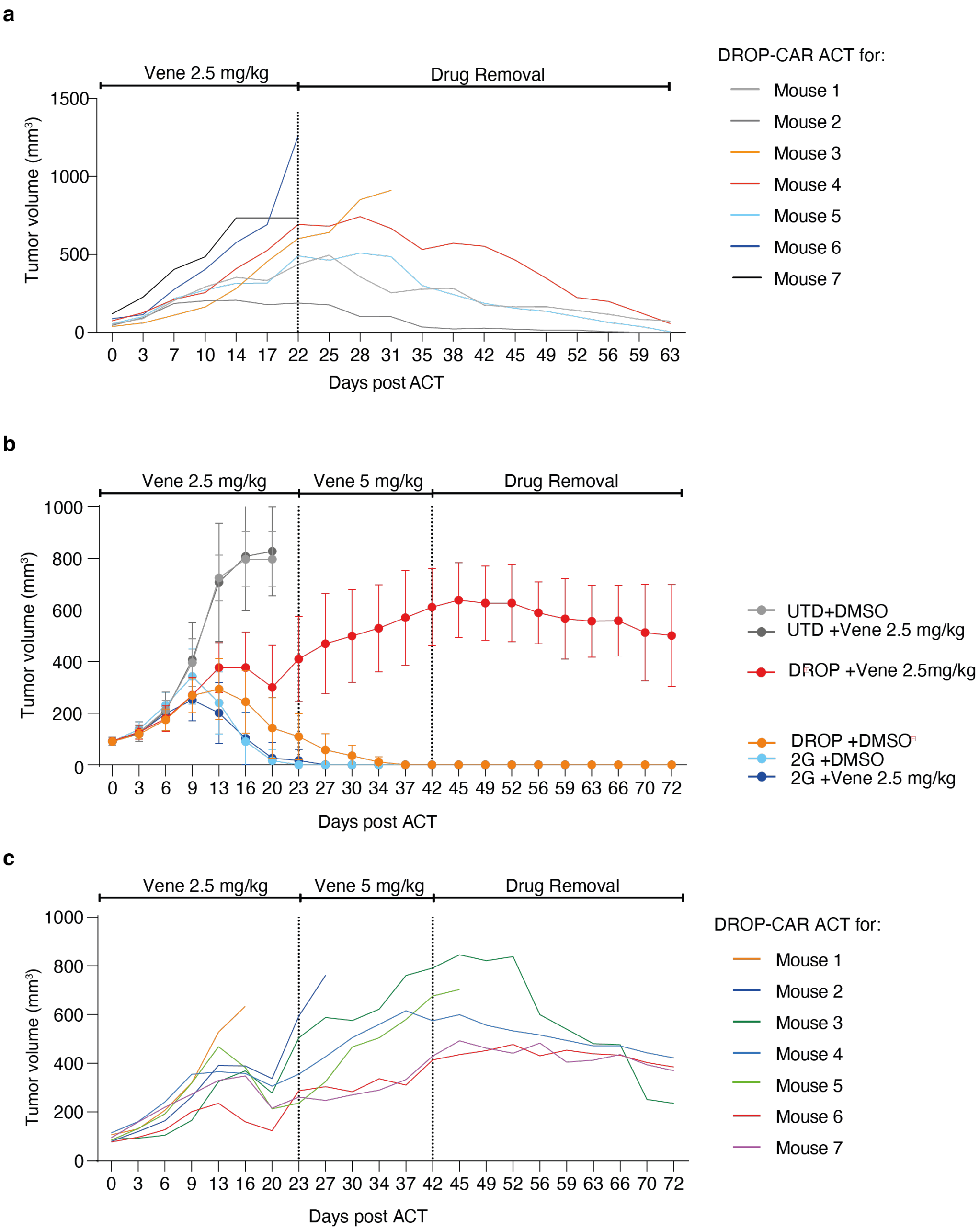

Supplementary Figure 9: Tumor control by DROP-CAR T cells.

a, Tumor growth curves of individual mice are shown for the DROP-CAR + venetoclax experimental group, before and after drug removal (Day 22). **b,** Replicate of the in vivo experiment for DROP-CAR T cells. In this experiment, at day 20 post ACT we observed incomplete repression of DROP-CAR activity in the DROP-CAR + venetoclax group compared to the vehicle control and increased the dose of venetoclax from 2.5 to 5 mg/kg. We then ceased the venetoclax administration to monitor DROP-CAR T cell re-activation after 42 days of venetoclax exposure (this is 20 days longer compared to the 22 days of exposure in a). The graph presents the mean tumor volumes of 7 mice per group +/- S.D. **c,** Tumor growth of individual mice shown for the DROP-CAR + venetoclax experimental group for panel b. (Venetoclax; Vene)
