# Supplementary material for: Drug-controlled CAR T cells through the regulation of cell–cell interactions
Source: Nat Chem Biol. 2026 Feb 19;22(10):1654–65. doi: 10.1038/s41589-026-02152-x (PMC13612217; doi:10.1038/s41589-026-02152-x)
Supplement: Supplementary file 2 [file 41589_2026_2152_MOESM2_ESM.pdf]

nature portfolio

Corresponding author(s): Melita Irving, Bruno Correia

Last updated by author(s): Nov 18, 2025

Reporting Summary

Nature Portfolio wishes to improve the reproducibility of the work that we publish. This form provides structure for consistency and transparency in reporting. For further information on Nature Portfolio policies, see our Editorial Policies and the Editorial Policy Checklist.

Statistics

For all statistical analyses, confirm that the following items are present in the figure legend, table legend, main text, or Methods section.

n/a	Confirmed	
☐	☒	The exact sample size (*n*) for each experimental group/condition, given as a discrete number and unit of measurement
☐	☒	A statement on whether measurements were taken from distinct samples or whether the same sample was measured repeatedly
☐	☒	The statistical test(s) used AND whether they are one- or two-sided<br>*Only common tests should be described solely by name; describe more complex techniques in the Methods section.*
☒	☐	A description of all covariates tested
☒	☐	A description of any assumptions or corrections, such as tests of normality and adjustment for multiple comparisons
☐	☒	A full description of the statistical parameters including central tendency (e.g. means) or other basic estimates (e.g. regression coefficient) AND variation (e.g. standard deviation) or associated estimates of uncertainty (e.g. confidence intervals)
☒	☐	For null hypothesis testing, the test statistic (e.g. *F*, *t*, *r*) with confidence intervals, effect sizes, degrees of freedom and *P* value noted<br>*Give P values as exact values whenever suitable.*
☒	☐	For Bayesian analysis, information on the choice of priors and Markov chain Monte Carlo settings
☒	☐	For hierarchical and complex designs, identification of the appropriate level for tests and full reporting of outcomes
☒	☐	Estimates of effect sizes (e.g. Cohen's *d*, Pearson's *r*), indicating how they were calculated

Our web collection on statistics for biologists contains articles on many of the points above.

Software and code

Policy information about availability of computer code

Data collection	Pymol and ChimeraX were used for structural visualization<br>Rosetta was used for protein design.<br>IncuCyte Instrument, BD LSR II FACS, BD LSR SORP FACS
Data analysis	Graphpad Prism 10, Microsoft Excel 16, FlowJo 10, IncuCyte Zoom 2016A Data analysis (Essen Bioscience), FACS DIVA Software, Biorender

For manuscripts utilizing custom algorithms or software that are central to the research but not yet described in published literature, software must be made available to editors and reviewers. We strongly encourage code deposition in a community repository (e.g. GitHub). See the Nature Portfolio guidelines for submitting code & software for further information.

Data

Policy information about availability of data

All manuscripts must include a data availability statement. This statement should provide the following information, where applicable:

- Accession codes, unique identifiers, or web links for publicly available datasets
- A description of any restrictions on data availability
- For clinical datasets or third party data, please ensure that the statement adheres to our policy

The data supporting the findings of this study are available within the article and its Supplementary Information.

Human research participants

Policy information about studies involving human research participants and Sex and Gender in Research.

Reporting on sex and gender	*Use the terms sex (biological attribute) and gender (shaped by social and cultural circumstances) carefully in order to avoid confusing both terms. Indicate if findings apply to only one sex or gender; describe whether sex and gender were considered in study design whether sex and/or gender was determined based on self-reporting or assigned and methods used. Provide in the source data disaggregated sex and gender data where this information has been collected, and consent has been obtained for sharing of individual-level data; provide overall numbers in this Reporting Summary. Please state if this information has not been collected. Report sex- and gender-based analyses where performed, justify reasons for lack of sex- and gender-based analysis.*
Population characteristics	*Describe the covariate-relevant population characteristics of the human research participants (e.g. age, genotypic information, past and current diagnosis and treatment categories). If you filled out the behavioural & social sciences study design questions and have nothing to add here, write "See above."*
Recruitment	*Describe how participants were recruited. Outline any potential self-selection bias or other biases that may be present and how these are likely to impact results.*
Ethics oversight	*Identify the organization(s) that approved the study protocol.*

Note that full information on the approval of the study protocol must also be provided in the manuscript.

Field-specific reporting

Please select the one below that is the best fit for your research. If you are not sure, read the appropriate sections before making your selection.

☒ Life sciences ☐ Behavioural & social sciences ☐ Ecological, evolutionary & environmental sciences

For a reference copy of the document with all sections, see nature.com/documents/nr-reporting-summary-flat.pdf

Life sciences study design

All studies must disclose on these points even when the disclosure is negative.

Sample size	For HEK293T and Jurkat cell culture experiments, n=3 biological replicates were predicted to be sufficient for estimating the spread of data, for calculating the mean and for detecting statistically significant differences between compared groups. Preliminary results and experience in similar cell culture experiments support n=3 biologically independent samples per individual experiment as sufficient to detect meaningful differences in reporter gene expression.<br>For the human T cells in vitro studies number of healthy donors and/or technical replicates were chosen according to the complexity of the assay and for the expected biological variability. For the in vivo studies maximum of 2 millions CAR T cells/mouse were needed. We achieved a sample size of 6-7 animal per treatment group which proved to be sufficient to reproducibly observe statistically significant differences.
Data exclusions	For the in vivo studies, upon calipering outlier mice with extreme burdens (either too high or too low compared to the average) were excluded from the experiment before CAR T cell transfer. No mice were excluded afterwards at any point.
Replication	All attempts at replication were successful. Anti-tumor activity could vary between T cell donors, both in vitro and in vivo.
Randomization	Tumor burden was evaluated by calipering the same day of CAR T cell transfer. Outlier mice with extreme burdens (either too high or too low compared to the average) were excluded from the experiment before CAR T cell transfer. No mice were excluded afterwards at any point. Following tumor burden measure, mice were assigned into treatment groups such that each group had the same overall average tumor volume. Buffy coats and apheresis filters were obtained from anonymous donors.
Blinding	To minimize stress to the mice, treatment administration and tumor volume measurements were made at the same time (during same anaesthesia). An independent investigator verified caliper measurements in a blinded fashion. Analysis of data (plotting of pre-recorded tumor volumes at end of study) was performed in a non-blinded manner.

Reporting for specific materials, systems and methods

We require information from authors about some types of materials, experimental systems and methods used in many studies. Here, indicate whether each material, system or method listed is relevant to your study. If you are not sure if a list item applies to your research, read the appropriate section before selecting a response.

Materials & experimental systems

n/a	Involved in the study
☐	☒ Antibodies
☐	☒ Eukaryotic cell lines
☒	☐ Palaeontology and archaeology
☐	☒ Animals and other organisms
☒	☐ Clinical data
☒	☐ Dual use research of concern

Methods

n/a	Involved in the study
☒	☐ ChIP-seq
☐	☒ Flow cytometry
☒	☐ MRI-based neuroimaging

Antibodies

Antibodies used

Antibodies were titrated for optimal staining. Near-IR fluorescent reactive dye (APC Cy-7) (L34976, Invitrogen) was used to assess viability. The following fluorophore conjugated antibodies were used for phenotypic memory analysis: BV711 mouse-anti-human CD3 (clone UCHT1, cat 563725, BD, Bioscience); BV605 mouse-anti-human CD4 (clone OKT4, cat 317438, Biolegend); APC-Cy7-labeled anti-human CD8 (clone SK1, cat A94683, Beckman Coulter); PE-Texas red-labeled mouse-anti-human CD45RA (clone HI100, cat B49193, Beckman Coulter); BV421 mouse-anti-human CCR7 (clone G043H7, cat 353208, Biolegend). AlexaFluo 647-conjugated anti-mouse F(ab)' (cat 115-606-072,Jackson Immuno Research) was used to evaluate the expression of the anti PSMA 2G, DEG and DROP CAR on primary T cells. AlexaFluo 647-conjugated anti-humanF(ab)' (cat 109-607-008, LubioScience) was used to evaluate the expression of the anti EpCAM 2G, DEG and DROP CAR on primary T cells. PE-labeled anti-human PSMA (clone LNI-17, cat 342504, lot no B211500, Biolegend) and PE Isotype mouse-anti-IgG1, k chain (clone MOPC-21, cat 400114, lot no B202580, Biolegend) were used to evaluated PSMA expression on cell line. Anti-human CD25-APC, CD71-PE and LAG3-PercpCy5.5 (cat 302610, 334106, 369312 respectively, Biolegend) were used to evaluate activatory ad inhibitory receptors induction upon drug administration.

Validation

The antibodies were the same routinely used in many previous publications, including our own, and were tested with appropriate positive and negative controls, as indicated in the experiments.

Eukaryotic cell lines

Policy information about cell lines and Sex and Gender in Research

Cell line source(s)

293T, Jurkat from ATCC. PC3 and PC3-PIP from Dr. Rosato, University of Padova.

Authentication

COA provided with cell line by ATCC. Properties pertinent to experiment (e.g. PSMA expression) were confirmed by flow cytometry.

Mycoplasma contamination

Cell lines were tested for mycoplasma contaminatoin and found negative.

Commonly misidentified lines
(See ICLAC register)

none

Animals and other research organisms

Policy information about studies involving animals; ARRIVE guidelines recommended for reporting animal research, and Sex and Gender in Research

Laboratory animals

NSG male mice, 8-12 weeks old, were bred and housed in a SOPF animal facility.

Wild animals

N/A

Reporting on sex

N/A

Field-collected samples

N/A

Ethics oversight

All the experiments have been conducted under the approval of cantonal veterinary office with the approved license 3570.

Note that full information on the approval of the study protocol must also be provided in the manuscript.

Flow Cytometry

Plots

Confirm that:

☒ The axis labels state the marker and fluorochrome used (e.g. CD4-FITC).

☒ The axis scales are clearly visible. Include numbers along axes only for bottom left plot of group (a 'group' is an analysis of identical markers).

☒ All plots are contour plots with outliers or pseudocolor plots.

☐ A numerical value for number of cells or percentage (with statistics) is provided.

Methodology

Sample preparation	Primary human T cells were isolated from the peripheral blood mononuclear cells (PBMCs) of healthy donors (HDs; prepared as buffycoats or apheresis filters). All blood samples were collected with informed consent of the HDs, and genetically-engineered with Ethics Approval from the Canton of Vaud to the laboratory of Dr. Coukos. Total PBMCs were obtained via Lymphoprep (Axonlab) separation solution, using a standard protocol of centrifugation. CD4+ and CD8+ T cells were isolated using a magnetic bead-based negative selection kit following the manufacturer's recommendations (easySEP, Stem Cell technology). Purified CD4+ and CD8+ T cells were cultured at a 1:1 ratio in RPMI-1640 with Glutamax, supplemented with 10% heat-inactivated FBS, 100 U/mL penicillin, 100 µg/mL streptomycin sulfate, and stimulated with anti-CD3 and anti-CD28 monoclonal antibody (mAb)-coated-beads (Lifetechnologies) in a ratio of 1:2, T cells: beads. For staining preparation, cells were washed once and resuspended in FACS buffer containing LIVE/DEAD dye and the antibody cocktail. Cells were incubated at 4 degrees for 30 minutes and washed twice before acquisition. Cells were not fixed prior to acquisition.
Instrument	LSR II, BD
Software	Collection: FACS DIVA<br>Analysis: FlowJo X
Cell population abundance	T cell purification from PBMCs by magnetic beads was validated by flow for CD4+/CD8+ cells. T cell purity was >99%.
Gating strategy	Starting cell population was gated on a linear SSC-A/FSC-A plot. Single cells were discriminated on a linear FSC-H/FSC-A plot. Live cells were determined by exclusion from positive Live/Dead stained cells. Positive/Negative populations were determined with negative controls.

☐ Tick this box to confirm that a figure exemplifying the gating strategy is provided in the Supplementary Information.
